# Cross-cultural validity of the Death Reflection Scale during the COVID-19 pandemic

**DOI:** 10.3389/fpsyg.2022.957177

**Published:** 2022-08-03

**Authors:** Christina Ramsenthaler, Klaus Baumann, Arndt Büssing, Gerhild Becker

**Affiliations:** ^1^School of Health Professions, Institute of Health Sciences, Zurich University of Applied Sciences, Winterthur, Switzerland; ^2^Cicely Saunders Institute of Palliative Care, Policy and Rehabilitation, King’s College London, London, United Kingdom; ^3^Wolfson Palliative Care Research Centre, Hull York Medical School, University of Hull, Hull, United Kingdom; ^4^Caritas Science and Christian Social Work, Faculty of Theology, University of Freiburg, Freiburg im Breisgau, Germany; ^5^IUNCTUS – Competence Center for Christian Spirituality, Philosophical-Theological Academy, Münster, Germany; ^6^Chair of Quality of Life, Spirituality and Coping, Faculty of Health, Witten/Herdecke University, Herdecke, Germany; ^7^Clinic for Palliative Medicine, University Medical Center Freiburg, Faculty of Medicine, University of Freiburg, Freiburg im Breisgau, Germany

**Keywords:** COVID-19 pandemic, death awareness, death reflection, life satisfaction, measurement invariance, cross-cultural validity

## Abstract

**Background:**

The global COVID-19 pandemic confronts people with their fragility, vulnerability, and mortality. To date, scales to measure death awareness mainly focus on the anxiety-provoking aspect of mortality cues. This study aims to cross-culturally adapt and validate the Death Reflection Scale (DRS), a scale for measuring positive, growth-oriented cognitions of life reflection and prosocial behavior following confrontation with the finiteness of life.

**Materials and Methods:**

The Death Reflection Scale was translated and adapted in a multi-step process to the German language. In this anonymous, cross-sectional, online survey at a large university in Germany, students, healthcare professionals (HCP) and other staff completed the DRS alongside comparison measures. Multi-group confirmatory factor analysis was used to assess configural, metric, and scalar measurement equivalence across four age and occupational groups. Convergent/divergent validity testing was done via Spearman correlations.

**Results:**

1,703 participants provided data for a response rate of ∼5%. 24% of respondents were HCP, 22% students. Confirmatory factor analysis showed a higher-order structure of the DRS with a strong general factor and the originally proposed five subscales (CFI 0.945, SRMR 0.045, RMSEA 0.055). Multi-group CFA showed partial metric equivalence across age groups and partial scalar invariance across occupational groups. Non-invariant scales were the *Motivation to live*, *Putting life into perspective*, and *Legacy* subscales. In the convergent validity testing, two hypotheses were fully confirmed, two partially and four were not confirmed. Experiencing a propensity for increased contemplation and life reflection during the pandemic together with spirituality showed correlations of moderate to large size to the DRS and its subscales (Spearman’s rho ranging from 0.31 to 0.52).

**Conclusion:**

Further conceptual work for death awareness to explore the construct’s stability in different population groups needs to be undertaken. However, the DRS can be mostly used to assess positive and growth-oriented aspects of death awareness and death reflection which may be an important avenue when developing counseling and support interventions for groups experiencing a high burden during the pandemic.

## Introduction

Since the Spanish Flu in 1918, there has been no other virus disease in the past century with such a wide spread and such high an impact on all societies around the globe like SARS-CoV-2. COVID-19 poses a direct threat to people’s life, health, economic welfare and thus their bio-psycho-social wellbeing (Bao et al., 2020). The pandemic confronts people in modern society with their fragility, vulnerability and ultimately their own mortality, either by being directly affected through infection, the infection of family members and friends or COVID-related death anxiety. While the past months have shown the widespread adoption of public health measures and vaccine development to control this infectious disease, prediction models point toward the persistence of SARS-CoV-2 as an endemic virus with seasonal epidemic peaks (Skegg et al., 2021; Telenti et al., 2021). COVID-19 presents as a disease with which societies have to learn to live, thus demonstrating the need to address the psychological aftermath of the pandemic (Heath et al., 2020; Skegg et al., 2021).

Epidemics and pandemics have been associated with detrimental consequences for the mental health of individuals, especially for healthcare professionals (HCP; Maunder et al., 2006; Batra et al., 2020; Schwartz et al., 2020; Temsah et al., 2020). Recent social surveys also demonstrated the high negative impact on adolescent and young adults or the university student population (Cielo et al., 2021; Kohls et al., 2021; Matos Fialho et al., 2021; Voltmer et al., 2021). Meta-analytical evidence points toward a high prevalence of post-traumatic symptoms and disorders for anxiety, depression, stress, post-traumatic stress syndrome, and burnout (Salari et al., 2020; Fan et al., 2021; Kunzler et al., 2021; Salehi et al., 2021; Zhao et al., 2021). However, these adverse effects on psychological health do not always occur after traumatic experiences (Tedeschi and Calhoun, 2004; Ramos and Leal, 2013; Hyun et al., 2021). Positive emotional states and growth with a quick recovery from trauma have been linked to constructs such as resilience, benefit finding (Tedeschi and Calhoun, 2004), meaning making (Park et al., 2008), and associated coping strategies (Tedeschi and Calhoun, 1996, 2004). Within the German context, positive changes during the pandemic in terms of a stronger reflection on personal priorities, goals, life philosophy as well as a higher awareness of relationships have been reported in various cancer and general population samples (Büssing et al., 2020a,b, 2021a,b,c).

Anxiety-provoking mortality cues as present in the pandemic may therefore not always lead to negative adaptive reactions in the individual. Contrary to the prevailing Terror Management Theory within social psychology (Solomon et al., 1991), which focuses solely on negative cognitions following mortality cues leading to self-protective actions and intergroup conflict via worldview defense (Greenberg et al., 1990; Solomon et al., 1991; Curşeu et al., 2021), researchers have also pointed toward the positive and growth-oriented mindset of death contemplation and reflection which may also foster prosocial actions (Yuan et al., 2019). To specifically study cognitions that may buffer the negative effects of death-related cues, Yuan et al. (2019) developed the Death Reflection Scale, a measure to determine positive aspects of death awareness, reflecting on life in relation to its finite nature like in the Stoic memento mori philosophy. The Death Reflection Scale thus opposes the main tenets of Terror Management Theory, namely that death-related cognitions are always anxiety-provoking and will lead to self-protective actions; also postulating that prosocial actions through death awareness are possible (Cozzolino et al., 2004; Cozzolino, 2006; Lykins et al., 2007; Grant and Wade-Benzoni, 2009). The scale has been validated in students, firefighters (Yuan et al., 2019) and the general population (Curşeu et al., 2021) where it has been found to measure aspects of positive death awareness distinct from death anxiety and life satisfaction (Yuan et al., 2019; Curşeu et al., 2021).

While instruments for measuring the death anxiety aspect are available in the German language (Klug, 1997), there is no similar death awareness/reflection scale available. The scale would help explain the positive changes documented in a variety of populations during the COVID-19 pandemic (Büssing et al., 2020a,b, 2021a,b,c) and thus provide a tool for growth-oriented interventions. Comparison measures for validity assessment were taken from these research studies due to their COVID-specific nature and their equilibrium in eliciting positive as well as negative changes/aspects. Furthermore, we chose indicators to measure the mortality salience of COVID-19 for each individual (i.e., witnessing deaths due to COVID-19 infections among family and friends) to understand whether death reflection is correlated with death awareness during the pandemic. Given the samples involved in the initial validation of the Death Reflection Scale in pre-pandemic years, the replicability of the latent construct and its potentially positive consequences in specialist workforces which experienced a direct confrontation with COVID-19 related deaths needs to be tested to further develop its incremental validity. Specifically, we are interested in whether the death reflection scale with its positive construct of death awareness shows invariance for age and occupational groups as has been found for the related construct of death anxiety according to terror management theory (Cicirelli, 2002; Pierce et al., 2007; Russac et al., 2007; Grant and Wade-Benzoni, 2009; Chopik, 2017; Zhong et al., 2021; Zampella and Benau, 2022).

The present study aims to (1) translate and adapt the Death Reflection Scale from English into German and explore its cross-cultural comparability and face validity, (2) evaluate the validity and reliability of the German version of the Death Reflection Scale in a large sample of university members and healthcare professionals, and (3) determine its measurement invariance across age and occupational groups.

## Materials and methods

### Translation and modification procedure

This study used a multi-step, explorative and cross-sectional study design. The translation and cultural adaptation followed commonly accepted standards as proposed by international outcome measures associations (Wild et al., 2005; Kulis et al., 2017). Several questionnaires focusing on death anxiety following mortality cues have been developed within the German language context (for example, Klug, 1997), thus providing wording options as a basis for the translation. The six-step process of translation was therefore shortened to the forward-backward translation procedure with subsequent expert review, omitting cognitive interviews. Instead, we focused on psychometric testing of equivalence in a large sample comprising occupational groups not included in the original validation study (Yuan et al., 2019).

Two independent, native German speakers from different backgrounds (palliative care/psychology and philosophy/theology, both proficient and fluent in English) forward translated the Death Reflection Scale. Two native English speakers with a nursing and epidemiology background, blinded to the original English version, backtranslated the scale. All discrepancies were discussed with a third independent researcher not involved in the translation at both stages. A record of all items and aspects challenging equivalence was kept and discussed in the subsequent expert review. The multidisciplinary focus group (*n* = 5) included experts from palliative care, theology, rehabilitation psychology and nursing to discuss cultural equivalence of key concepts. The topic guide was based on the Cultural Equivalence Model for Translating and Adapting Instruments (Chavez and Canino, 2005), comprising conceptual, content, semantic and technical aspects of equivalence. Changes made during this process were specified according to the criteria proposed by Koller et al. (2012). The consolidated version of the Death Reflection Scale was then pre-tested in ten survey participants, with further alterations to phrasing being made for two items. The final version was included in the survey for psychometric testing.

### Participants and procedures

This validation is part of a larger, single-center, prospective online survey research project focusing on wellbeing and perceived changes in university students and staff during the Coronavirus pandemic (see study details in the German Registry of Clinical Studies, no. DRKS00023789). A convenience sample was recruited from March to June 2021 during the third wave of the COVID-19 pandemic in Germany. All study measures were administered via the online survey tool platform LimeSurvey^®^ (Version 3.22.27) (LimeSurvey Project Team, 2021). Inclusion criteria were: age ≥18 years, being a registered student or working with at least a part-time contract at the university or one of its affiliated institutions, and possessing an up-to-date email address (as an indicator of membership) and being sufficiently fluent in German to complete the survey. This study was conducted according to principles outlined in the Declaration of Helsinki 2013 (Mastroleo, 2016). The Ethics Committee of Albert-Ludwigs-Universität Freiburg, Germany (No. 20-1160) approved the study. The survey was completely anonymously with only categorized socio-demographic details being collected.

### Measures

#### Sociodemographic data and information on coronavirus infections

Sociodemographic data, including age group, gender, religious affiliation, university department and occupational group, living situation (being partnered/not living alone, living alone), and whether or not respondents had children were collected. The infection status of each participant and whether deaths due to Coronavirus disease had been observed among family, friends or acquaintances were asked at the beginning of the survey.

#### Death Reflection Scale

The Death Reflection Scale is a 15-item measure with items eliciting how people reflect on life in relation to its finite nature. The five subscales (a) *Motivation to help* (altruistic and prosocial, helpful behavior, i.e., “When I think about death, I feel a strong urge to help other people”), (b) *Motivation to live* (pursuing goals in life, trying new things; i.e., “When I think about death, I make plans for my life”), (c) *Putting life in perspective* (a more relaxed attitude toward stressful or irritating experiences; i.e., “When I think about death, I can let go of the little problems”), (d) leaving a personal *Legacy* (i.e., “When I think about death, I reflect on how I will be remembered”), and (e) *Connection to others* (spending time and expressing feelings toward loved ones and friends; i.e., “When I think about death, I want to spend more time with the people I care about”). (Dis)agreement is rated on a six-point scale. The DRS was initially validated in a sample of students and firefighters and showed good psychometric properties (Yuan et al., 2019). Cronbach’s alpha ranged from 0.73 to 0.87 for the five subscales (Yuan et al., 2019). The five-factor model provided a better fit than a model with a higher-order factor subsuming the five factors despite six of the ten correlations among latent factors being in the high range (*r* > 0.50) (Yuan et al., 2019). The lowest correlations among the five dimensions range from 0.14 (between *Putting life into* perspective and *Legacy*) to 0.48 (between *Legacy* and *Connection to others*). A subsequent confirmatory factor analysis of the DRS has used a higher-order factor model to compute an average item score (Curşeu et al., 2021).

#### Life satisfaction and wellbeing

Life satisfaction was measured with the 12-item Brief Multidimensional Life Satisfaction Scale (BMLSS; Büssing et al., 2009, 2020b), assessing satisfaction with different areas of life (personal life, friends and social life, work life, general situation, financial situation, and health) on a seven-point (dis)agreement scale. It has been validated in a sample of older and chronically ill adults (Büssing et al., 2009), showing good internal consistency, convergent and discriminant validity. Cronbach’s alpha for the scale is 0.87.

Subjective well-being was assessed via the five-item generic World Health Organization Well-being Index (WHO-5; Bech et al., 2003), a measure derived from mental health questionnaires and scales. The five items are: “Over the past 2 weeks, (1) I have felt cheerful and in good spirits; (2) I have felt calm and relaxed; (3) I have felt active and vigorous; (4) I woke up feeling refreshed and rested; (5) my daily life has been filled with things that interest me.” All items are rated on a 6-point scale. Raw sum scores can be converted to a 0–100 (maximum wellbeing) scale. The validity, responsiveness and diagnostic validity of the WHO-5 have been demonstrated in several studies (Bech et al., 2003; Löwe et al., 2004; Krieger et al., 2014; Topp et al., 2015). Cronbach’s alpha for the WHO-5 is 0.85.

#### COVID-19 related stressors and perceived changes

Stressors during the COVID-19 pandemic in the form of perceived restrictions in daily life, feeling under pressure or stressed, feeling anxious or insecure, feeling lonely or socially isolated and being worried due to one’s financial-economic situation were assessed via five numerical rating scales ranging from 0 – “not at all” to 100 – “very strong” (Büssing et al., 2020a). These scales have been shown to have good internal consistency and good convergent validity (Büssing et al., 2020a). Cronbach’s alpha was 0.78.

Changes in attitudes and behaviors during the pandemic were assessed via a 35-item modified Perception of Change Questionnaire (PCQ; Büssing et al., 2020a,b, 2021c). Items in the PCQ focus on changes in the following areas: (a) Nature/Silence/Contemplation (7 items, Cronbach’s alpha: 0.83); (b) Spirituality (4 items of originally 5 items, Cronbach’s alpha: 0.87); (c) Relationships (5 items from the original 6 items, Cronbach’s alpha: 0.77); (d) Reflection on life (3 items; Cronbach’s alpha: 0.68); (e) Digital media usage (3 items; Cronbach’s alpha: 0.77); (f) Restrictions in life (5 items instead of the original 3, Cronbach’s alpha: 0.77). The validity of the questionnaire has been demonstrated in a series of studies (Büssing et al., 2020a,b, 2021c).

The measure was extended by a 7-item new subscale “Memento mori” (Cronbach’s alpha: 0.83) including items of COVID-related death anxiety with feelings of insecurity due to witnessed deaths, feeling unsettled by the high number of deaths, being afraid of catching the disease, or thinking about one’s own death in the pandemic. We aimed to capture specific cognitive/appraisal (i.e., worry, perceived threat), and emotional (i.e., anxiety) aspects based on the psychological anxiety and coping literature (Lazarus, 1993; Ohman, 2000). Following the distinction between state and trait, the items refer to an acute feeling of unpleasant arousal and not measuring the general disposition of fear of death and dying. Due to the lack of COVID-related death anxiety scales at the time of designing the study, we adapted items from available death anxiety questionnaires (emotional aspects, Templer et al., 1971; Neimeyer, 1997/1998) coupled with COVID-specific mortality salience aspects (Sliter et al., 2014; Rossi et al., 2020). At the time of study design, no COVID-specific mortality salience scale had been developed. This 7-item subscale has not been tested for validity/reliability.

#### Indicators of spirituality

The seven-item Awe/Gratitude scale (GrAw-7) measures the perceptive aspects of secular spirituality (nature or specific situations inspiring awe and subsequent feelings of gratitude) that have been found to be relevant to less or non-religious persons (Büssing et al., 2018). All items are scored on a 4-point scale ranging from 0 – “never” to 3 – “regularly,” combined for a total transformed sum score (maximum: 100). This scale has been shown to have good psychometric properties (Büssing et al., 2018). Cronbach’s alpha was 0.84.

### Statistical analysis

All statistical analyses were done in R v.4.0.4 (R Core Team, 2020) and mainly used the lavaan (Rosseel, 2012) and psych packages (Revelle, 2020).

Sociodemographic and psychological measures are presented descriptively according to age and occupational groups. We tried to replicate the originally proposed factor model (Yuan et al., 2019) in a series of nested confirmatory factor models (CFA) using random draws from the sample. Given the ordered and non-normal nature of the items in the DRS, we used the Yuan-Bentler estimator with robust standard errors and additionally bootstrapped fit statistics (Rosseel, 2012). Due to exclusion of withdrawals and the nature of the survey setup, there were no missing data. We contrasted the original five-factor model with a model with correlated errors and a higher-order factor analytical model (Curşeu et al., 2021). Goodness-of-fit indices were: Comparative Fit Index (CFI, <0.090), the Tucker-Lewis Index (TLI, <0.090), Root Mean Square Error of Approximation (RMSEA, <0.05), and the Standardized Root Mean Square Residual (SRMR, <0.08) (Hu and Bentler, 1999). Competing models were also compared via Akaike’s Information Criterion (AIC) and the Bayesian Information Criterion (BIC), with smaller values indicating comparatively better fit.

Measurement invariance across age and occupational groups was tested via multi-group CFAs (Horn and McArdle, 1992; Meredith, 1993; Byrne, 2008, 2012; Brown, 2015). This four-step procedure poses increasingly restrictive assumptions on measurement equivalence. First, configural equivalence as the basic level of invariance explores whether the construct has the same meaning (factorial structure) in different groups. Next, metric equivalence tests constraints on factor loadings to be equal across group to establish a common metric. Scalar invariance hypothesizes that both factor loadings and intercepts are equal across groups. The highest level of invariance is found with strict invariance which requires factor loadings, intercepts, and residual variances to be invariant across groups. Partial invariance may be found by relaxing some constraints when some non-invariant parameters are encountered (Byrne et al., 1989). Nested models were compared via chi-square difference tests (Δ*X*^2^; Cheung and Rensvold, 2002; Byrne, 2012) and change in CFI (ΔCFI) of less than 0.01 (Cheung and Rensvold, 2002). The sample size of 1,703 is well over the recommended upper target of 1,000 participants as per Meade and Lautenschlager’s (2004) recommendations based on simulations exceeding 12 items in a scale. Due to small group size and the anonymized data collection of occupational groups in broad categories, measurement invariance testing for occupational groups only included students, researchers, HCPs, and administrative staff and excluding laboratory and other (unspecified) occupation.

Convergent/divergent validity was evaluated by Spearman correlations (non-normal and skewed distributions) and biserial rank correlations (for binary variables and DRS) with bootstrapped standard errors. We tested the following hypotheses:

1. (H1a and b) Personal COVID-19 infection or having witnessed related deaths will show a small positive correlation to the DRS total score and all subscale scores (Curşeu et al., 2021) (convergent validity).
2. (H2) Female gender will be positively moderately associated with *Motivation to help*, *Motivation to live*, and *Putting life into perspective* (Mazza et al., 2020; Curşeu et al., 2021; Hyun et al., 2021) (convergent validity).
3. (H3) COVID-related death anxiety will be positively and at least moderately associated with the DRS total score and all subscale scores (Yuan et al., 2019; Curşeu et al., 2021) (convergent validity).
4. (H4) Perceiving restrictions in daily life as a burden will be negatively associated with the DRS total score and its subscale scores (Curşeu et al., 2021) (discriminant validity).
5. (H5) Death reflection will be negatively moderately associated with items measuring negative mood or stress (Curşeu et al., 2021) (discriminant validity).
6. (H6) Reporting more contemplation and reflection on life due to COVID-19 will be positively moderately associated with the DRS and its subscales (Büssing et al., 2020a,b, 2021a,b,c) (convergent validity).
7. (H7) Spirituality and reporting faith as a source of support will be positively associated with the DRS total score, *Motivation to help*, *Motivation to live*, and *Putting life into perspective* (convergent validity), but not to *Legacy* and *Connection to others* (Büssing et al., 2020b, 2021b) (discriminant validity).
8. (H8) Life satisfaction shows at least small positive correlations to the DRS total score and *Connection to others* (Yuan et al., 2019) (convergent validity), but not the other subscales (discriminant validity).

## Results

### Sample characteristics

Sociodemographic information is presented in Table 1. Of 2,083 respondents (out of 40,075 potential respondents at the university and its medical center), 1,703 provided complete responses to the Death Reflection Scale items. The response rate is 4.71%. Respondents were mostly female and under the age of 40. Twenty-two percent of the sample are students. Over 50% are HCP. When comparing the demographic information with reported distributions (Albert-Ludwigs University Freiburg, 2021), under-sampling was mainly present for students from all departments and for male administrative staff in the economics, natural sciences and medical departments. Over-sampling was present for female research and administration staff in all university departments.

**TABLE 1:** Socio-demographic information (*n* = 1,703).

	n	%
**Gender**		
Men	436	25.6
Women	1,267	74.4
Age group		
<30 years	631	37.1
31–40 years	346	20.3
41–50 years	282	16.6
51–60 years	342	20.1
>60 years	102	5.9
**Religious affiliation**		
Christian	1,054	61.9
Other	39	2.3
None	603	35.4
Missing	7	0.4
**Department/Faculty**		
Humanities	187	11.0
Behavioral sciences, legal studies, economics	104	6.1
Biology, chemistry, physics, environmental sciences	174	10.2
Technology, computer science	63	3.7
Medicine	943	55.4
Administration	187	11.0
Other university departments	42	2.5
Missing	3	0.2
**Occupational group**		
Student	372	21.8
Research	287	16.9
Healthcare professional	407	23.9
Administration, IT, support, library	426	25.0
Laboratory	92	5.4
Other	119	7.0
**Marital status**		
Living with partner	1,061	62.3
Living alone	580	34.1
Missing	62	3.6
**Children**		
Yes	704	41.3
Missing	1	0.1
**Tested for Coronavirus infection**		
Yes, positively tested	79	4.6
Yes, negatively tested	1,055	61.9
Not tested	569	33.5
Witnessed deaths due to Coronavirus		
Yes	336	19.7
Missing	11	0.6
**Irritated or unsettled by conflicting and inconsistent statements about danger/course of Coronavirus infections in the public media**		
Not at all	707	41.5
A little	694	40.8
Somewhat	261	15.3
Very much	41	2.4

Sociodemographic information as well as descriptive statistics on scales and subscales can be found in the Supplementary Appendix 1.

### Forward-backward translation

The completed and consolidated German translation of the Death Reflection Scale can be found in Supplementary Appendix 2. When reviewing discrepancies, problems were noted with item #8 (“I am able to stop sweating the small stuff”) and item #10 (“I think about what legacy I will have left behind”). Both items use English colloquialisms that do not have a direct correspondence in the Germany language. Both items were also discussed regarding their conceptual overlap with surrounding items from the respective subscale (i.e., item #7 “I can let go of the little problems” and item #8). Problems were also noted with translating the names of the *Putting life into perspective* and *Legacy* subscales into German. Consensus was reached on the final translation at the end of the expert review focus group with two more changes being made to item #7 and #8 in the pre-testing.

### Item statistics and distributional properties

Item and scale descriptives for the DRS total score and its subscales are shown in Tables 2, 3 (separately for age and occupational groups). Distributions of subscales across groups are shown in Supplementary Appendix 3, Supplementary Figures 1, 2. Further differences in subscales of measures other than the DRS among age and occupational groups are shown in Supplementary Appendix 4, 5. *Legacy* consistently shows the lowest scale means in the total and subsamples. *Connection to others* presents with the highest scale means, also across groups. Discrepancies in the item responses across age and occupational groups are present for subscales except for *Connection to others* in age groups.

**TABLE 2:** The Death Reflection Scale items and scale descriptives in different age groups.

	Total (*N* = 1,703)	Age <30 years (*n* = 631)	Age 31–40 years (*n* = 346)	Age 41–50 years (*n* = 282)	Age >50 years (*n* = 444)		
DRS subscales	*M (SD)*	*M (SD)*	*M (SD)*	*M (SD)*	*M (SD)*	*X* ^2^	*p*
Motivation to help	7.11 (4.2)	7.92 (4.1)	6.73 (4.1)	6.72 (4.1)	6.49 (4.2)	39.6	0.000
Skewness	−0.14	−0.31	−0.10	0.00	−0.02		
Kurtosis (SE)	−0.81 (0.1)	−0.65 (0.2)	−0.89 (0.2)	−0.75 (0.2)	−0.88 (0.2)		
Alpha	0.87	0.86	0.87	0.84	0.87		
Motivation to live	7.89 (4.1)	8.98 (3.8)	7.67 (4.3)	7.11 (3.8)	7.00 (4.0)	78.5	0.000
Skewness	−0.25	−0.47	−0.12	−0.18	−0.10		
Kurtosis (SE)	−0.68 (0.1)	−0.30 (0.2)	−0.85 (0.2)	−0.68 (0.2)	−0.79 (0.2)		
Alpha	0.84	0.82	0.88	0.80	0.82		
Putting life into perspective	6.09 (4.2)	5.31 (4.0)	5.79 (4.3)	6.59 (4.2)	7.13 (4.3)	55.8	0.000
Skewness	0.22	0.50	0.27	0.09	−0.12		
Kurtosis (SE)	−0.85 (0.1)	−0.54 (0.2)	−0.85 (0.2)	−0.82 (0.3)	−0.88 (0.2)		
Alpha	0.88	0.88	0.90	0.85	0.87		
Legacy	5.75 (4.5)	6.96 (4.5)	5.16 (4.3)	5.27 (4.3)	4.80 (4.2)	75.0	0.000
Skewness	0.37	0.02	0.68	0.44	0.59		
Kurtosis (SE)	−0.89 (0.1)	−1.05 (0.2)	−0.36 (0.2)	−0.77 (0.3)	−0.62 (0.2)		
Alpha	0.85	0.84	0.86	0.83	0.86		
Connection to others	10.44 (4.2)	10.75 (3.9)	10.50 (4.1)	10.48 (4.3)	9.94 (4.6)	4.6	0.201
Skewness	−0.96	−1.04	−0.94	−1.03	−0.78		
Kurtosis (SE)	0.13 (0.1)	0.61 (0.2)	0.11 (0.2)	0.21 (0.3)	−0.49 (0.2)		
Alpha	0.90	0.88	0.88	0.90	0.93		
Total score	7.46 (2.9)	7.98 (2.8)	7.17 (2.9)	7.23 (2.9)	7.07 (3.0)	33.4	0.000
Skewness	−0.50	−0.62	−0.36	−0.47	−0.45		
Kurtosis (SE)	−0.06 (0.1)	0.40 (0.1)	−0.42 (0.2)	0.00 (0.2)	−0.30 (0.1)		
Omega	0.74	0.69	0.70	0.71	0.70		

DRS, Death Reflection Scale; SD, standard deviation; SE, standard error; y, years.

**TABLE 3:** The Death Reflection Scale items and scale descriptives in different occupational groups.

	Total* (*N* = 1,492)	Student (*n* = 372)	Research (*n* = 287)	HCP (*n* = 407)	Admin (*n* = 426)		
DRS subscales	*M (SD)*	*M (SD)*	*M (SD)*	*M (SD)*	*M (SD)*	*X* ^2^	*p*
Motivation to help	7.19 (4.2)	8.32 (3.9)	6.68 (4.3)	6.75 (4.2)	6.95 (4.1)	38.5	0.000
Skewness	−0.15	−0.42	−0.12	0.01	−0.08		
Kurtosis (SE)	−0.8 (0.1)	−0.46 (0.2)	−0.99 (0.3)	−0.82 (0.2)	−0.78 (0.2)		
Alpha	0.88	0.88	0.89	0.85	0.89		
Motivation to live	7.90 (4.0)	8.97 (3.8)	7.20 (4.2)	7.66 (3.9)	7.68 (3.9)	36.9	0.000
Skewness	−0.26	−0.48	−0.27	−0.14	−0.15		
Kurtosis (SE)	−0.64 (0.1)	−0.34 (0.2)	−0.85 (0.3)	−0.64 (0.2)	−0.67 (0.2)		
Alpha	0.83	0.82	0.88	0.80	0.82		
Putting life into perspective	6.03 (4.2)	5.27 (4.1)	5.75 (4.2)	6.49 (4.3)	6.46 (4.3)	23.3	0.000
Skewness	0.23	0.54	0.23	0.08	0.11		
Kurtosis (SE)	−0.86 (0.1)	−0.45 (0.2)	−0.96 (0.3)	−0.93 (0.2)	−0.87 (0.2)		
Alpha	0.88	0.88	0.90	0.85	0.87		
Legacy	5.87 (4.5)	7.38 (4.5)	5.20 (4.4)	5.7 (4.5)	5.1 (4.2)	61.7	0.000
Skewness	0.36	−0.12	0.51	0.45	0.60		
Kurtosis (SE)	−0.91 (0.1)	−1.05 (0.2)	−0.72 (0.3)	−0.78 (0.2)	−0.53 (0.2)		
Alpha	0.86	0.84	0.86	0.83	0.86		
Connection to others	10.45 (4.2)	10.46 (3.9)	9.35 (4.6)	11.05 (4.2)	10.63 (4.0)	31.3	0.000
Skewness	−0.96	−0.91	−0.69	−1.18	−0.95		
Kurtosis (SE)	0.15 (0.1)	0.45 (0.2)	−0.6 (0.3)	0.61 (0.2)	0.12 (0.2)		
Alpha	0.90	0.88	0.88	0.90	0.93		
Total score	7.49 (2.9)	8.08 (2.8)	6.83 (3.1)	7.54 (2.8)	7.37 (2.8)	26.4	0.000
Skewness	−0.49	−0.51	−0.54	−0.52	−0.35		
Kurtosis (SE)	−0.04 (0.1)	0.12 80.1)	−0.37 (0.2)	0.02 80.19	−0.19 (0.1)		
Omega	0.70	0.70	0.74	0.66	0.69		

*The following occupational groups were omitted from this analysis: Laboratory staff (n = 92), other (belonging to another, not further specified occupational group; n = 119). Admin, Administrative, IT, library support staff; DRS, Death Reflection Scale; HCP, healthcare professional; SD, standard deviation; SE, standard error.

### Confirmatory factor analysis and measurement equivalence

Goodness-of-fit of CFA models is contrasted in Table 4. Standardized factor loadings for the final higher-order factor model are shown in Table 5, further information on models is shown in Supplementary Appendix 6. A unidimensional model (all 15 items loading on one factor) was contrasted with the original five-factor solution, a second-order model and a higher-order model (bifactor model: all items loading onto a general factor and loading onto their respective subscale with no correlations between subscales). Despite all models showing some extent of absolute misfit, the higher-order model showed the best relative, acceptable fit with an RMSEA of 0.055 (90% CI of 0.050, 0.060, not significant; recommended cut-off: <0.05 to 0.08), a CFI of 0.945 (recommended cut-off: >0.90), an SRMR of 0.045 (recommended cut-off: <0.08) and the lowest AIC and BIC values among the models. Factor loadings for the general factor ranged from 0.30 (item #9, “I am less stressed about the things that are bothering me”) to 0.66 (item #14, “I want to tell the people I care about how I feel about them”). Cronbach’s alpha was at a minimum of 0.84 for *Motivation to live*. The total score had an omega of 0.74. Alpha differed in age and occupational groups by a maximum of 10 decimal points (Tables 2, 3). Omega for the total score was poorest in those being 31–40 years old and in HCPs.

**TABLE 4:** Comparison of different factor analytical baseline models for the DRS.

	*X*^2^(*df*)	RMSEA	[90% CI]	CFI	SRMR	AIC	BIC
Unidimensional model	4242 (90)*	0.165	0.160, 0.169	0.407	0.147	88682	88845
5-factor model	598.2 (80)*	0.062	0.057, 0.066	0.926	0.039	80927	81144
Second-order model	618.4 (85)*	0.061	0.056, 0.065	0.924	0.051	80990	81181
Higher-order model (Bifactor model)	463.4 (75)*	0.055	0.050, 0.060	0.945	0.045	80814	81058

*X^2^ test statistically significant.

AIC, Akaike’s information criterion; BIC, Bayesian (Schwartz) information criterion; CFI, comparative fit index; CI, confidence interval; df, degrees of freedom; DRS, Death Reflection Scale; RMSEA, Root mean square error of approximation; SRMR, standardized root mean square residual.

**TABLE 5:** Confirmatory factor analysis for a higher-order factor solution for the total sample.

When I think about death….	*G*	*F1*	*F2*	*F3*	*F4*	*F5*
1. I feel like I should do more for the world.	0.54	0.54				
2. I feel a strong urge to help other people.	0.58	0.72				
3. I want to be a more generous person.	0.64	0.53				
4. I make plans for my life.	0.60		0.52			
5. I reflect on the things I still want to do.	0.65		0.57			
6. I am motivated to try new things.	0.58		0.44			
7. I can let go of the little problems.	0.39			0.77		
8. I am able to stop sweating the small stuff.	0.36			0.86		
9. I am less stressed about the things that are bothering me.	0.30			0.68		
10. I think about what legacy I will have left behind.	0.54				0.45	
11. I reflect on whether people will think of me after death.	0.39				0.81	
12. I reflect on how I will be remembered.	0.49				0.76	
13. I want to spend more time with the people I care about.	0.64					0.63
14. I want to tell the people I care about how I feel about them.	0.66					0.57
15. I want to spend more time with my family.	0.56					0.61
**Fit indices**						
Yuan-Bentler X^2^ (df)	463.4 (75)					
RMSEA [90% CI]	0.055 [0.050–0.060]					
CFI	0.945					
SRMR	0.045					

CFI, comparative fit index; CI, confidence interval; df, degrees of freedom; F1–F5, Factors 1–5; G, general factor; RMSEA, root mean square error of approximation; SRMR, standardized root mean square residual.

The results from multi-group equality testing are presented in Table 6. For both comparisons, full invariance was not reached. For the different age groups, full metric invariance (weakest level of invariance) with the construct having the same meaning across age groups was not reached (as indicated by the significant Yuan-Bentler *X*^2^ test). By freeing item #6 (“I am motivated to try new things”) on *Motivation to live*, partial metric invariance was obtained. Item #6 thus shows non-invariant factor loadings across age groups. Also freeing item #9 (“I am less stressed about the things that are bothering me,” *Putting life into perspective*) and item #12 (“I reflect on how I will be remembered,” *Legacy*) resulted in better model fit for partial metric equivalence. Item #6 had the lowest factor loading in 41–50-year-olds, the highest in the 50 + group. Item #9 had the lowest loading for <30-year-olds, the highest in 41–50-year-olds. Even with the less constrained model, partial scalar invariance could not be reached.

**TABLE 6:** Goodness-of-fit indices for measurement invariance models for the five-factor higher-order Death Reflection Scale.

*n* = 1,703	YB *X*^2^	*df*	X^2^/df	CFI	RMSEA [90% CI]	SRMR	ΔX^2^	Δdf	*p* < 0.01	ΔCFI	AIC
**Age group invariance (4 groups)**											
Model 1: Configural invariance	940.5	320	2.94	0.935	0.057 [0.051, 0.062]	0.044	–	–	–	–	80590
Model 2: Full metric invariance	993.6	350	2.84	0.936	0.049 [0.044, 0.054]	0.046	53.10	30	0.006	0.001	80583
Model 2a: Partial metric invariance	959.8	341	2.81	0.936	0.051 [0.046, 0.056]	0.043	19.29	21	No	0.001	80586
Model 3: Scalar invariance	1053.3	381	2.86	0.932	0.050 [0.045, 0.055]	0.046	93.52	30	0.000	0.004	80617
Model 3a: Partial scalar invariance	1035.3	381	2.72	0.932	0.052 [0.047, 0.057]	0.045	75.51	30	0.000	0.004	80621
Model 4: Strict invariance	1354.9	395	3.43	0.907	0.060 [0.055, 0.065]	0.046	267.86	14	0.000	0.016	80855
**Occupational group invariance**											
Model 1: Configural invariance	891.1	320	2.78	0.959	0.059 [0.053, 0.065]	0.043	–	–	–	–	70413
Model 2: Metric invariance	940.5	350	2.69	0.958	0.052 [0.047, 0.058]	0.045	49.39	30	No	0.001	70403
Model 3: Scalar invariance	1002.1	380	2.64	0.956	0.053 [0.048, 0.058]	0.047	61.60	30	0.004	0.002	70404
Model 3a: Partial scalar invariance	977.67	371	2.63	0.955	0.052 [0.047, 0.058]	0.046	37.17	21	No	0.003	70409
Model 4: Strict invariance	1186.5	395	3.00	0.943	0.073 [0.068, 0.078]	0.047	184.36	24	0.000	0.012	70559

AIC, Akaike’s information criterion; CFI, comparative fit index; df, degrees of freedom; RMSEA, root mean square error of approximation; SRMR, standardized root mean square residual; YB, Yuan Bentler X^2^.

For the different occupational groups, partial scalar invariance, a medium level of equivalence, was reached (see Table 6). While metric invariance (equal factor loadings across occupational groups) was present, freeing the intercepts of items #10, #11, #12 (*Legacy*) indicated students using the answer scale of these items in a different way. Equal item intercepts are necessary for assessing mean differences across groups which is only supported for four of the five subscales of the DRS (Model 3a).

### Convergent and discriminant validity

Table 7 documents the associations for convergent/divergent validity testing. Two hypotheses were confirmed (H6, H7), two partially confirmed (H3, H5), and four were not confirmed (H1, H2, H4, H8). Spirituality (H7) and a propensity for increased contemplation and reflection in life (H6) showed mostly moderate positive correlations as hypothesized (convergent validity). COVID-related death anxiety showed positive moderate correlations with the DRS except for *Putting life into perspective* (H3, convergent validity); but *Stressors* had only small and not moderate associations with the DRS (H5, convergent validity). A personal COVID-19 infection/witnessing deaths did not result in small correlations (H1, convergent validity), neither did life satisfaction or wellbeing (H8, convergent and discriminant validity). Female gender only showed small correlations (H2, convergent validity) and restrictions in life were mostly positively associated with the DRS (H4, discriminant validity).

**TABLE 7:** Convergent and discriminant validity testing for the Death Reflection Scale (*n* = 1,703).

	DRS total	Motivation to help	Motivation to live	Putting life into perspective	Legacy	Connection to others
Women	**0.215**	**0.124**	**0.201**	0.086	−0.051	***0.391***
COVID-19 infection	0.131	−0.020	−0.056	**0.247**	0.133	0.138
Deaths family	0.026	0.011	−0.083	0.001	0.073	0.079
PCQ-Memento mori	***0.386***	**0.298**	**0.219**	**0.101**	***0.361***	***0.348***
Restricted in daily life	0.066	**0.092**	**0.131**	−**0.147**	0.063	**0.091**
PCQ-Restrictions	**0.134**	**0.153**	**0.168**	−**0.171**	**0.137**	**0.178**
Under pressure	0.062	**0.084**	**0.088**	−**0.207**	**0.103**	**0.147**
Anxious	**0.167**	**0.181**	**0.145**	−**0.159**	**0.229**	**0.178**
Lonely	**0.099**	**0.093**	**0.145**	−**0.165**	**0.156**	**0.113**
Financial worries	0.075	0.078	0.086	−0.066	0.081	0.079
PCQ-N/S/C	***0.420***	**0.286**	***0.306***	***0.439***	**0.125**	***0.304***
PCQ-Reflection	***0.423***	***0.354***	***0.339***	**0.083**	***0.331***	***0.354***
PCQ-Relationships	***0.374***	**0.287**	**0.283**	**0.261**	**0.117**	***0.353***
Gratitude/awe	**0.299**	**0.232**	**0.184**	***0.323***	0.085	**0.214**
Faith as support	***0.359***	**0.251**	**0.059**	***0.519***	**0.133**	**0.275**
PCQ-Spirituality	**0.297**	**0.263**	**0.146**	**0.212**	**0.190**	**0.212**
Life satisfaction	0.048	−0.004	0.026	**0.244**	−**0.107**	0.012
Well-being	0.073	0.073	0.052	***0.300***	−0.076	−0.023

Bolded coefficients: p < 0.05; bolded coefficients in italics: at least moderate effect size r > 0.30 (Cohen, 1988). DRS, Death Reflection Scale; N/S/C, Nature/Silence/contemplation subscale of the Perceived Changes Questionnaire; PCQ, Perceived Changes Questionnaire.

## Discussion

This study translated, cross-culturally adapted, and examined the validity of the Death Reflection Scale across different groups and in comparison, to related constructs. The Death Reflection Scale is a measure of positive death awareness and death-related cognitions eliciting the extent of a positive and growth mindset when being confronted with mortality cues as are common in the COVID-19 pandemic. The 15-item scale was found to exhibit a higher-order factor structure with one general factor and five distinct subscales. Thus, we were able to replicate findings by Curşeu et al. (2021) who also proposed forming a total scale score based on high loadings of all items on a general factor. The different factor structure to the original validation may also stem from cultural differences in the construct death reflection in our German sample. This aspect could be explored in further research. The low yet acceptable relative fit of the model which does not clear the more stringent thresholds of a CFI > 0.950 (Hu and Bentler, 1999) may be explained by the low number of three items per subscale which may be close to empirical under-identification in some cases (Byrne, 2012; Brown, 2015). However, measurement invariance testing further shows the heterogeneous validity of the DRS in subsamples (Byrne et al., 1989; Meredith, 1993).

The results regarding configural factorial measurement invariance across groups indicate that participants of different age and occupational backgrounds (students, researchers, healthcare professionals, and administrative staff) use an identical cognitive framework when processing death awareness and reflection. However, only partial metric invariance was achieved across age groups with factor loadings on *Motivation to live*, *Putting life into perspective*, and *Legacy* needing to be freed. This indicates that items contribute differently to the latent construct across ages. Overall, higher factor loadings for the invariant subscales were observed for older age groups. This may indicate mortality cues having a different saliency across the lifespan (Carstensen et al., 1999; Specht et al., 2011; Reed et al., 2014). This result of higher death reflection in those aged 50 years or older reverses the negative association between age and death anxiety, the negative affective reaction when presented with mortality cues as posited in Terror Management Theory (Cicirelli, 2002; Pierce et al., 2007; Russac et al., 2007; Grant and Wade-Benzoni, 2009; Chopik, 2017; Zhong et al., 2021; Zampella and Benau, 2022). It could also point toward the two constructs of death anxiety and death reflection being more closely linked in latent profile groups as has been shown by Zhong et al. (2021). The authors also demonstrated that membership in a profile group systematically varied across occupational groups depending on the perceived relevance of COVID-19 as a mortality cue.

For occupational groups, while the construct may be understood similarly in different professions, partial scalar invariance points toward the Likert-type answer scale being used in a non-identical way for items 10–12 (*Legacy*). It remains debatable whether full scalar equivalence needs to be present for meaningful comparisons across different groups (Davidov et al., 2014). Invariance will affect the unbiased estimation of latent means across groups, thus precluding a direct comparison of the mean scores (Meuleman, 2012; Oberski, 2014). Partial equivalence is supported when there are at least two indicators per construct that are invariant at the metric and scalar levels to allow cross-group comparisons (Byrne et al., 1989), which is the case for four out of five subscales of the DRS. Furthermore, non-equivalence for the *Legacy* subscale may be confounded by non-equivalence across age groups (see above).

Overall, HCPs often presented with comparatively low latent scores on the subscales whereas students estimated the highest latent scores on *Motivation to help*, *Motivation to live*, and *Legacy*. This finding is contrary to effects found in the original validation studies where these aspects showed comparatively higher means in a sample of firefighters (Yuan et al., 2019). The authors also reported mortality cues being positively related to death reflection. They explain their finding by this group being subject to frequent mortality cues over a longer period of time which allows them to transcend their death-related anxiety. The comparatively lower scores in our sample for the group being presented with the most direct mortality cues may represent a self-selection mechanism of more resilient staff already engaging in meaning-making activities taking part. Tendencies of highly burdened staff quitting their job during the pandemic have been reported for the German healthcare system (Röthke et al., 2021). Students, on the other hand, have been identified as the population group experiencing maybe the starkest alteration of their normal lifestyle with online teaching, social restrictions and financial-economic insecurity due to the pandemic (Cielo et al., 2021; Kohls et al., 2021; Matos Fialho et al., 2021; Voltmer et al., 2021). Despite these multiple stressors in students’ life, answers on the Death Reflection Scale may also indicate this group’s higher propensity to engage in meaning-making and post-traumatic growth with more changes in death reflection taking place (Herbert et al., 2021; Kohls et al., 2021).

The convergent/discriminant validity testing of the Death Reflection Scale in our German sample mostly showed only a partial confirmation of proposed relationships. Only two hypotheses for convergent validity were confirmed, the remaining six hypotheses for convergent and discriminant validity were only partially or not confirmed. The largest contrasts to the original validation study and further research by Curşeu et al. (2021) were found regarding the role of life satisfaction and COVID-related death anxiety or negative cognitions regarding death and dying. We failed to observe the small correlations with life satisfaction reported by Yuan et al. (2019) in our data. The authors proposed a mediating role for life satisfaction and the relationship between death anxiety and death reflection. We also were not able to corroborate the hypothesis that death reflection is negatively associated with negative mood as found by Curşeu et al. (2021). These results could either point toward measurement bias with our measures or could mean that those experiencing high COVID-related stressors and emotional burden may also be those engaging in more meaning-making cognitions. This result needs further exploration. The buffering effect of spirituality was also shown in a recent study by Büssing et al. (2021b) who demonstrated more positive changes in those reporting more gratitude and awe (a secular aspect of spirituality).

The current study is limited by several factors, with the non-probabilistic sample from a single center likely resulting in sampling bias. Moreover, while the response rate of almost 5% is considered reasonable for student or university surveys (Ramm, 2014), based on free-text comments in the survey under-sampling of intensive care unit staff and oversampling of respondents with a higher psychological burden may have occurred. During the data collection time, the area in Germany from which we sampled was – despite local lockdown measures being in place – not among the most severely hit regions during the third wave. While this can certainly be perceived as a positive development, it may also have resulted in less mortality cues being present in people’s daily life, leading to restrictions being more acutely and severely perceived as stressful. Furthermore, we did not use measures of death anxiety validated and used in comparison studies (Yuan et al., 2019; Curşeu et al., 2021), but developed our own instrument with death anxiety being elicited related to COVID-19. While this approach may have had a higher saliency with regard to the pandemic, this subscale has not been fully validated and thus may introduce measurement bias into the results, thus attenuating correlations (Lord et al., 1968). However, to our knowledge no current Coronavirus anxiety scale (i.e., Lee, 2020) measures the mortality salience aspect of the pandemic. In addition to non-equivalence across age and occupational groups, we had also initially planned to investigate gender invariance of the DRS. However, due to severe undersampling of men in the administrative, healthcare professional and student group, we thought this bias to severe to do so.

The Death Reflection Scale may present the first scale within the German language context to measure positive aspects of death-related cognition and awareness. In the current pandemic with a high level of stressors, frequent mortality cues and the danger of adverse effects on mental health of groups particularly affected by the pandemic, the scale may present the opportunity to assess positive changes in relation to these stressors, in the form of a greater awareness when confronted with the finiteness of life. The scale may help in the implementation of interventions to foster such cognitions, resilience, adaptive coping, and post-traumatic growth by pointing toward positive aspects such as greater connectedness to others, altruism, and reflection on life goals. These aspects may help when processing traumatic and highly stressful events and may help create a growth environment shifting from the negative impact to the potential of meaning making and transcendence.

## Data availability statement

The raw data supporting the conclusions of this article will be made available by the authors, without undue reservation. According to the data protection regulations at the Medical Research Centre, the raw data supporting the conclusions of this article are only available from the corresponding author upon reasonable request.

## Ethics statement

The studies involving human participants were reviewed and approved by the Ethics Committee of Albert-Ludwigs-Universität Freiburg, Germany (No. 20–1160). Study participants provided consent by opting into the survey. Information about the study was available on the first page as a downloadable file. The survey itself was anonymous. All procedures performed in the study are in accordance with the ethical standards of the Institutional and National Research Committee and with the 1964 Helsinki Declaration and its later amendments. Written informed consent for participation was not required for this study in accordance with the national legislation and the institutional requirements.

## Author contributions

CR provided the original translation, collected and analyzed the data, interpreted the results, and wrote the first draft of the manuscript. AB and KB designed the original instruments used for convergent and discriminant validity testing. AB, KB, and GB commented on the revised Death Reflection Scale and revised the manuscript critically. All authors contributed to the article and approved the submitted version.

## References

[B1] Albert-Ludwigs University Freiburg (2021). *Jahresbericht des Rektors 2019-2020 [Annual Report of the Principal and President 2019-2020].* Breisgau: University of Freiburg.

[B2] Bao Y. Sun Y. Meng S. Shi J. Lu L. (2020). 2019-nCoV epidemic: address mental health care to empower society. *Lancet* 395 e37–e38. 10.1016/S0140-6736(20)30309-3 32043982 PMC7133594

[B3] Batra K. Singh T. P. Sharma M. Batra R. Schvaneveldt N. (2020). Investigating the psychological impact of COVID-19 among healthcare workers: a meta-analysis. *Int. J. Environ. Res. Public Health* 17:9096. 10.3390/ijerph17239096 33291511 PMC7730003

[B4] Bech P. Olsen L. R. Kjoller M. Rasmussen N. K. (2003). Measuring well-being rather than the absence of distress symptoms: a comparison of the SF-36 mental health subscale and the WHO-five well-being scale. *Int. J. Methods Psychiatr. Res.* 12 85–91. 10.1002/mpr.145 12830302 PMC6878541

[B5] Brown T. A. (2015). *Confirmatory Factor Analysis for Applied Research.* New York, NY: Guilford.

[B6] Büssing A. Fischer J. Haller A. Heusser P. Ostermann T. Matthiessen P. F. (2009). Validation of the brief multidimensional life satisfaction scale in patients with chronic diseases. *Eur. J. Med. Res.* 14 171–177. 10.1186/2047-783x-14-4-171 19380290 PMC3401007

[B7] Büssing A. Hübner J. Walter S. Gießler W. Büntzel J. (2020a). Tumor patients’ perceived changes of specific attitudes, perceptions, and behaviors due to the COVID-19 pandemic and its relation to reduced wellbeing. *Front. Psychiatry* 11:574314. 10.3389/fpsyt.2020.574314 33192703 PMC7581913

[B8] Büssing A. Rodrigues Recchia D. Hein R. Dienberg T. (2020b). Perceived changes of specific attitudes, perceptions and behaviors during the Corona pandemic and their relation to wellbeing. *Health Qual. Life Outcomes* 18:374. 10.1186/s12955-020-01623-6 33256755 PMC7702679

[B9] Büssing A. Recchia D. R. Baumann K. (2018). Validation of the awe/gratitude questionnaire and its association with disposition of gratefulness. *Religions* 9:117.

[B10] Büssing A. Recchia D. R. Hübner J. Walter S. Büntzel J. Büntzel J. (2021a). Tumor patients’ fears and worries and perceived changes of specific attitudes, perceptions and behaviors due to the COVID-19 pandemic are still relevant. *J. Cancer Res. Clin. Oncol.* 147 1673–1683. 10.1007/s00432-021-03573-y 33675401 PMC7936591

[B11] Büssing A. Rodrigues Recchia D. Dienberg T. Surzykiewicz J. Baumann K. (2021b). Awe/gratitude as an experiential aspect of spirituality and its association to perceived positive changes during the COVID-19 pandemic. *Front. Psychiatry* 12:642716. 10.3389/fpsyt.2021.642716 33959049 PMC8095710

[B12] Büssing A. Rodrigues Recchia D. Dienberg T. Surzykiewicz J. Baumann K. (2021c). Dynamics of perceived positive changes and indicators of well-being within different phases of the COVID-19 pandemic. *Front. Psychiatry* 12:685975. 10.3389/fpsyt.2021.685975 34211412 PMC8239218

[B13] Byrne B. (2012). *Structural Equation Modeling with Mplus: Basic Concepts, Applications, and Programming.* New York, NY: Routledge.

[B14] Byrne B. M. (2008). Testing for multigroup equivalence of a measurement instrument: a walk through the process. *Psicothema* 20 872–882. 18940097

[B15] Byrne B. M. Shavelson R. J. Muthén B. (1989). Testing for the equivalence of factor covariance and mean structures: the issue of partial measurement invariance. *Psychol. Bull.* 105 456–466.

[B16] Carstensen L. L. Isaacowitz D. M. Charles S. T. (1999). Taking time seriously: a theory of socioemotional selectivity. *Am. Psychol.* 54 165–181. 10199217 10.1037//0003-066x.54.3.165

[B17] Chavez L. Canino G. (2005). *Toolkit on Translating and Adapting Instruments.* Cambridge, MA: Human Services Research Institute.

[B18] Cheung G. W. Rensvold R. B. (2002). Evaluating goodness-of-fit indexes for testing measurement invariance. *Struct. Equ. Model.* 9 233–255.

[B19] Chopik W. J. (2017). Death across the lifespan: age differences in death-related thoughts and anxiety. *Death Stud.* 41 69–77. 10.1080/07481187.2016.1206997 27573253

[B20] Cicirelli V. G. (2002). Fear of death in older adults: predictions from terror management theory. *J. Gerontol. Ser. B Psychol. Sci. Soc. Sci.* 57 358–366. 10.1093/geronb/57.4.p358 12084786

[B21] Cielo F. Ulberg R. Di Giacomo D. (2021). Psychological impact of the COVID-19 outbreak on mental health outcomes among youth: a rapid narrative review. *Int. J. Environ. Res. Public Health* 18:6067. 10.3390/ijerph18116067 34199896 PMC8200066

[B22] Cohen J. (1988). *Statistical Power Analysis for the Behavioral Sciences.* Hillsdale, NJ: Lawrence Erlbaum Associates.

[B23] Cozzolino P. J. (2006). Death contemplation, growth, and defense: converging evidence of dual-existential systems? *Psychol. Inq.* 17 278–287.

[B24] Cozzolino P. J. Staples A. D. Meyers L. S. Samboceti J. (2004). Greed, death, and values: from terror management to transcendence management theory. *Pers. Soc. Psychol. Bull.* 30 278–292. 15030620 10.1177/0146167203260716

[B25] Curşeu P. L. Coman A. D. Panchenko A. Fodor O. C. Raţiu L. (2021). Death anxiety, death reflection and interpersonal communication as predictors of social distance towards people infected with COVID 19. *Curr. Psychol.* [Epub ahead of print]. 10.1007/s12144-020-01171-8 33686325 PMC7930891

[B26] Davidov E. Meuleman B. Cieciuch J. Schmidt P. Billiet J. (2014). Measurement equivalence on cross-national research. *Annu. Rev. Sociol.* 40 55–75.

[B27] Fan F. C. Zhang S. Y. Cheng Y. (2021). Incidence of psychological illness after coronavirus outbreak: a meta-analysis study. *J. Epidemiol. Commun. Health* 75 836–842. 10.1136/jech-2020-215927 33632722 PMC7908057

[B28] Grant A. M. Wade-Benzoni K. A. (2009). The hot and cool of death awareness at work: mortality cues, aging, and self-protective and prosocial motivations. *Acad. Manag. Rev.* 34 600–622.

[B29] Greenberg J. Pyszczynski T. Solomon S. Rosenblatt A. Veeder M. Kirkland S. (1990). Evidence for terror management theory II: the effects of mortality salience on reactions to those who threaten or bolster the cultural worldview. *J. Pers. Soc. Psychol.* 58 308–318.

[B30] Heath C. Sommerfield A. von Ungern-Sternberg B. S. (2020). Resilience strategies to manage psychological distress among healthcare workers during the COVID-19 pandemic: a narrative review. *Anaesthesia* 75 1364–1371. 10.1111/anae.15180 32534465 PMC7323405

[B31] Herbert C. Bolock A. E. Abdennadher S. (2021). How do you feel during the COVID-19 pandemic? A survey using psychological and linguistic self-report measures, and machine learning to investigate mental health, subjective experience, personality, and behaviour during the COVID-19 pandemic among university students. *BMC Psychol.* 9:90. 10.1186/s40359-021-00574-x 34078469 PMC8170461

[B32] Horn J. L. McArdle J. J. (1992). A practical and theoretical guide to measurement invariance in aging research. *Exp. Aging Res.* 18 117–144. 1459160 10.1080/03610739208253916

[B33] Hu L.-T. Bentler P. M. (1999). Cut off criteria for fit indexes in covariance structure analysis: conventional criteria versus new alternatives. *Struct. Equ. Model.* 6 1–55.

[B34] Hyun H. S. Kim M. J. Lee J. H. (2021). Factors associated with post-traumatic growth among healthcare workers who experienced the outbreak of MERS virus in South Korea: a mixed-method study. *Front. Psychol.* 12:541510. 10.3389/fpsyg.2021.541510 33967871 PMC8100316

[B35] Klug A. (1997). *Einstellungen zu Sterben, Tod und Danach [Attitudes Towards Dying, Death and the Hereafter Questionnaire].* Aachen: Verlag Mainz.

[B36] Kohls E. Baldofski S. Moeller R. Klemm S.-L. Rummel-Kluge C. (2021). Mental health, social and emotional well-being, and perceived burdens of university students during COVID-19 pandemic lockdown in Germany. *Front. Psychiatry* 12:643957. 10.3389/fpsyt.2021.643957 33889102 PMC8055863

[B37] Koller M. Kantzer V. Mear I. Zarzar K. Martin M. Greimel E. (2012). The process of reconciliation: evaluation of guidelines for translating quality-of-life questionnaires. *Expert Rev. Pharmacoecon. Outcomes Res.* 12 189–197. 10.1586/erp.11.102 22458620

[B38] Krieger T. Zimmermann J. Huffziger S. Ubl B. Diener C. Kuehner C. (2014). Measuring depression with a well-being index: further evidence for the validity of the WHO Well-Being Index (WHO-5) as a measure of the severity of depression. *J. Affect. Disord.* 156 240–244. 10.1016/j.jad.2013.12.015 24412323

[B39] Kulis D. Bottomley A. Velikova G. Greimel E. Koller M. (2017). *EORTC Quality of Life Group Translation Procedure Manual.* Available Online at: https://www.eortc.org/app/uploads/sites/2/2018/02/translation_manual_2017.pdf (accessed May 30, 2022).

[B40] Kunzler A. M. Röthke N. Günthner L. Stoffers-Winterling J. Tüscher O. Coenen M. (2021). Mental burden and its risk and protective factors during the early phase of the SARS-CoV-2 pandemic: systematic review and meta-analyses. *Global. Health* 17:34. 10.1186/s12992-021-00670-y 33781283 PMC8006628

[B41] Lazarus R. S. (1993). “Why we should think of stress as a subset of emotion,” in *Handbook of Stress. Theoretical and Clinical Aspects*, eds Goldberger L. Bretznitz S. (New York, NY: The Free Press), 217–239.

[B42] Lee S. A. (2020). Coronavirus anxiety scale: a brief mental health screener for COVID-19 related anxiety. *Death Stud.* 44 393–401. 10.1080/07481187.2020.1748481 32299304

[B43] LimeSurvey Project Team (2021). *LimeSurvey: An Open Source Survey Tool.* Hamburg: LimeSurvey.

[B44] Lord F. M. Novick M. R. Birnbaum A. (1968). *Statistical Theory of Mental Test Scores.* New York, NY: Addison-Wesley.

[B45] Löwe B. Spitzer R. L. Gräfe K. Kroenke K. Quenter A. Zipfel S. (2004). Comparative validity of three screening questionnaires for DSM-IV depressive disorders and physicians’ diagnoses. *J. Affect. Disord.* 78 131–140. 10.1016/s0165-0327(02)00237-9 14706723

[B46] Lykins E. L. Segerstrom S. C. Averill A. J. Evans D. R. Kemeny M. E. (2007). Goal shifts following reminders of mortality: reconciling posttraumatic growth and terror management theory. *Pers. Soc. Psychol. Bull.* 33 1088–1099. 17578931 10.1177/0146167207303015

[B47] Mastroleo I. (2016). Post-trial obligations in the declaration of Helsinki 2013: classification, reconstruction and interpretation. *Dev. World Bioeth.* 16 80–90. 10.1111/dewb.12099 26481322

[B48] Matos Fialho P. M. Spatafora F. Kühne L. Busse H. Helmer S. M. Zeeb H. (2021). Perceptions of study conditions and depressive symptoms during the COVID-19 pandemic among university students in Germany: results of the international COVID-19 student well-being study. *Front. Public Health* 9:674665. 10.3389/fpubh.2021.674665 34178930 PMC8222519

[B49] Maunder R. G. Lancee W. J. Balderson K. E. Bennett J. P. Borgundvaag B. Evans S. (2006). Long-term psychological and occupational effects of providing hospital healthcare during SARS outbreak. *Emerg. Infect. Dis.* 12 1924–1932. 10.3201/eid1212.060584 17326946 PMC3291360

[B50] Mazza C. Ricci E. Biondi S. Colasanti M. Ferracuti S. Napoli C. (2020). A nationwide survey of psychological distress among Italian people during the COVID-19 pandemic: immediate psychological responses and associated factors. *Int. J. Environ. Res. Public Health* 17:3165. 10.3390/ijerph17093165 32370116 PMC7246819

[B51] Meade A. W. Lautenschlager G. J. (2004). A Monte-Carlo study of confirmatory factor analytic tests of measurement equivalence/invariance. *Struct. Equ. Model.* 11 60–72.

[B52] Meredith W. (1993). Measurement invariance, factor analysis and factorial invariance. *Psychometrika* 58 525–543.

[B53] Meuleman B. (2012). “When are intercept differences substantively relevant in measurement invariance testing,” in *Methods, Theories and Empirical Applications in the Social Sciences: Festschrift for Peter Schmidt*, eds Salzborn S. Davidov E. Reinecke J. (Wiesbaden: Springer VS), 97–104.

[B54] Neimeyer R. A. (1997/1998). Death anxiety research: the state of the art. *Omega* 36 97–120.

[B55] Oberski D. L. (2014). Evaluating sensitivity of parameters of interest to measurement invariance in latent variable models. *Polit. Anal.* 22 45–60.

[B56] Ohman A. (2000). “Fear and anxiety: evolutionary, cognitive, and clinical perspectives,” in *Handbook of Emotions*, eds Lewis M. Haviland-Jones J. M. (New York, NY: Guilford), 573–593.

[B57] Park C. L. Edmondson D. Fenster J. R. Blank T. O. (2008). Meaning making and psychological adjustment following cancer: the mediating roles of growth, life meaning, and restored just-world beliefs. *J. Consult. Clin. Psychol.* 76 863–875. 10.1037/a0013348 18837603

[B58] Pierce J. D. Cohen A. B. Chambers J. A. Meade R. M. (2007). Gender differences in death anxiety and religious orientation among US high school and college students. *Ment. Health Relig. Cult.* 10 143–150.

[B59] R Core Team (2020). *R: A Language and Environment for Statistical Computing. Version 4.0.3.* Vienna: R Foundation for Statistical Computing.

[B60] Ramm M. (2014). Response, Stichprobe und Repräsentativität: zwei Dokumentationen zum deutschen Studierendensurvey [Response, sample and representativeness: two documentations for the German student survey]. *Hefte zur Bildungs- und Hochschulforschung* 72 1–56.

[B61] Ramos C. Leal I. (2013). Posttraumatic growth in the aftermath of trauma: a literature review about related factors and application contexts. *Psychol. Commun. Health* 2 43–54.

[B62] Reed A. E. Chan L. Mikels J. A. (2014). Meta-analysis of the age-related positivity effect: age differences in preferences for positive over negative information. *Psychol. Aging* 29 1–15. 24660792 10.1037/a0035194

[B63] Revelle W. (2020). *psych: Procedures for Personality and Psychological Research. Version 2.0.12.* Available Online at: https://CRAN.R-project.org/package=psych (accessed May 30, 2022).

[B64] Rosseel Y. (2012). lavaan: an R package for structural equation modeling. *J. Stat. Softw.* 48 1–36.

[B65] Rossi A. Panzeri A. Pietrabissa G. Manzoni G. M. Castelnuovo G. Mannarini S. (2020). The anxiety-buffer hypothesis in the time of COVID-19: when self-esteem protects from the impact of loneliness and fear on anxiety and depression. *Front. Psychol.* 11:2177. 10.3389/fpsyg.2020.02177 33240140 PMC7683508

[B66] Röthke N. Wollschläger D. Kunzler A. M. Rohde A. Molter S. Bodenstein M. (2021). Psychische belastung, resilienz und absentismusneigung bei gesundheitspersonal in deutschland während der ersten COVID-19-pandemiewelle im Frühjahr 2020: eine Ad-hoc-Befragung [Mental burden, resilience and tendency towards absenteeism among healthcare personnel in Germany during the first wave of the COVID-19 pandemic in spring 2020: an ad hoc survey]. *Nervenarzt* 92 579–590. 10.1007/s00115-021-01132-x 34009438 PMC8132043

[B67] Russac R. J. Gatliff C. Reece M. Spottswood D. (2007). Death anxiety across the adult years: an examination of age and gender effects. *Death Stud.* 31 549–561. 17726829 10.1080/07481180701356936

[B68] Salari N. Hosseinian-Far A. Jalali R. Vaisi-Raygani A. Rasoulpoor S. Mohammadi M. (2020). Prevalence of stress, anxiety, depression among the general population during the COVID-19 pandemic: a systematic review and meta-analysis. *Global. Health* 16:57. 10.1186/s12992-020-00589-w 32631403 PMC7338126

[B69] Salehi M. Amanat M. Mohammadi M. Salmanian M. Rezaei N. Saghazadeh A. (2021). The prevalence of post-traumatic stress disorder related symptoms in Coronavirus outbreaks: a systematic-review and meta-analysis. *J. Affect. Disord.* 282 527–538. 10.1016/j.jad.2020.12.188 33433382 PMC7831964

[B70] Schwartz R. Sinskey J. L. Anand U. Margolis R. D. (2020). Addressing postpandemic clinician mental health: a narrative review and conceptual framework. *Ann. Intern. Med.* 173 981–988. 10.7326/M20-4199 32822206 PMC7450528

[B71] Skegg D. Gluckman P. Boulton G. Hackmann H. Karim S. S. A. Piot P. (2021). Future scenarios for the COVID-19 pandemic. *Lancet* 397 777–778. 10.1016/S0140-6736(21)00424-4 33607000 PMC7906624

[B72] Sliter M. T. Sinclair R. R. Yuan Z. Mohr C. D. (2014). Don’t fear the reaper: trait death anxiety, mortality salience, and occupational health. *J. Appl. Psychol.* 99 759–769. 10.1037/a0035729 24490968

[B73] Solomon S. Greenberg J. Pyszczynski T. (1991). “A terror management theory of social behavior: the psychological function of self-esteem and cultural worldviews,” in *Advances in Experimental Social Psychology*, ed. Zanna M. (San Diego, CA: Academic Press), 91–159.

[B74] Specht J. Egloff B. Schmukle S. C. (2011). Stability and change of personality across the life course: the impact of age and major life events on mean-level and rank-order stability of the Big Five. *J. Pers. Soc. Psychol.* 101 862–882. 21859226 10.1037/a0024950

[B75] Tedeschi R. G. Calhoun L. G. (1996). The posttraumatic growth inventory: measuring the positive legacy of trauma. *J. Trauma. Stress* 9 455–471. 10.1007/BF02103658 8827649

[B76] Tedeschi R. G. Calhoun L. G. (2004). Posttraumatic growth: conceptual foundations and empirical evidence. *Psychol. Inq.* 15 1–18.

[B77] Telenti A. Arvin A. Corey L. Corti D. Diamond M. S. García-Sastre A. (2021). After the pandemic: perspectives on the future trajectory of COVID-19. *Nature* 596 495–504. 10.1038/s41586-021-03792-w 34237771

[B78] Templer D. I. Ruff C. F. Franks C. M. (1971). Death anxiety - age, sex, and parental resemblance in diverse populations. *Dev. Psychol.* 4:108.

[B79] Temsah M.-H. Al-Sohime F. Alamro N. Al-Eyadhy A. Al-Hasan K. Jamal A. (2020). The psychological impact of COVID-19 pandemic on health care workers in a MERS-CoV endemic country. *J. Infect. Public Health* 13 877–882. 10.1016/j.jiph.2020.05.021 32505461 PMC7256548

[B80] Topp C. W. Østergaard S. D. Søndergaard S. Bech P. (2015). The WHO-5 Well-Being Index: a systematic review of the literature. *Psychother. Psychosom.* 84 167–176. 10.1159/000376585 25831962

[B81] Voltmer E. Köslich-Strumann S. Walther A. Kasem M. Obst K. Kötter T. (2021). The impact of the COVID-19 pandemic on stress, mental health and coping behavior in German University students - a longitudinal study before and after the onset of the pandemic. *BMC Public Health* 21:1385. 10.1186/s12889-021-11295-6 34256717 PMC8275908

[B82] Wild D. Grove A. Martin M. Eremenco S. McElroy S. Verjee-Lorenz A. (2005). Principles of good practice for the translation and cultural adaptation process for patient-reported outcomes (PRO) measures: report of the ISPOR task force for translation and cultural adaptation. *Value Health* 8 94–104. 10.1111/j.1524-4733.2005.04054.x 15804318

[B83] Yuan Z. Baranik L. E. Sinclair R. R. Sliter M. T. Rand K. L. Salyers M. P. (2019). *Memento mori*: the development and validation of the death reflection scale. *J. Organ. Behav.* 40 417–433.

[B84] Zampella B. J. Benau E. M. (2022). Delay of gratification, gender role attitudes, and death reflections predict death anxiety. *Omega* [Epub ahead of print]. 10.1177/00302228221085177 35445615

[B85] Zhao Y.-J. Jin Y. Rao W.-W. Li W. Zhao N. Cheung T. (2021). The prevalence of psychiatric comorbidities during the SARS and COVID-19 epidemics: a systematic review and meta-analysis of observational studies. *J. Affect. Disord.* 287 145–157. 10.1016/j.jad.2021.03.016 33799032 PMC7948672

[B86] Zhong R. Paluch R. M. Shum V. Zatzick C. D. Robinson S. L. (2021). Hot, cold, or both? A person-centered perspective on death awareness during the COVID-19 pandemic. *J. Appl. Psychol.* 106 839–855. 10.1037/apl0000931 34138590

