# Supplementary material for: Cross-cultural validity of the Death Reflection Scale during the COVID-19 pandemic
Source: Front Psychol. 2022 Aug 3;13:957177. doi: 10.3389/fpsyg.2022.957177 (PMC9382129; doi:10.3389/fpsyg.2022.957177)
Supplement: Supplementary file 1 [file Data_Sheet_1.docx]

Supplemental Appendix to

Cross-cultural validity of the Death Reflection Scale during the COVID-19 pandemic

Christina Ramsenthaler^1,2,3^, Klaus Baumann^4,6^, Arndt Büssing^5,6^, Gerhild Becker^7*^

^1^School of Health Professions, Institute of Health Sciences, Zurich University of Applied Sciences ZHAW, 8401 Winterthur, Switzerland

^2^Cicely Saunders Institute of Palliative Care, Policy and Rehabilitation, King’s College London, Bessemer Road, London SE5 9PJ, United Kingdom

^3^Wolfson Palliative Care Research Centre, Hull York Medical School, University of Hull, Allam Medical Building, Hull HU6 7RX, United Kingdom

^4^Caritas Science and Christian Social Work, Faculty of Theology, University of Freiburg, 79085 Freiburg, Germany

^5^Chair of Quality of Life, Spirituality and Coping, Faculty of Health, University of Witten/Herdecke, 58313 Herdecke, Germany

^6^IUNCTUS – Competence Center for Christian Spirituality, Philosophical-Theological Academy, 48149 Münster, Germany

^7^Clinic for Palliative Medicine, Medical Center – University of Freiburg, Faculty of Medicine, University of Freiburg, 79106 Freiburg, Germany

*** Correspondence:**Prof Dr Dipl-Theol Dipl-Caritaswiss Gerhild Becker MSc, Clinic for Palliative Medicine, Medical Center – University of Freiburg, Robert-Koch-Str. 3, 79106 Freiburg, Germany, Phone: +49 (0)761 270 95411, [gerhild.becker@uniklinik-freiburg.de](mailto:gerhild.becker@uniklinik-freiburg.de)

**Online Appendix 1: Description of scales used for convergent/discriminant validity testing**

**Online Table 1** Descriptive statistics for scales and items other than the Death Reflection Scale (*n* = 1,703)

|  | **n** | **%** |  |  |
| --- | --- | --- | --- | --- |
| *COVID-19 own infection* |  |  |  |  |
| Yes | 79 | 4.6 |  |  |
| No | 1,624 | 95.4 |  |  |
| *Witnessed deaths among family/friends due to Coronavirus* |  |  |  |  |
| Yes | 336 | 19.7 |  |  |
| No | 1,356 | 80.3 |  |  |
| *Faith as a source of support* |  |  |  |  |
| Yes | 219 | 12.9 |  |  |
| Partly | 499 | 29.3 |  |  |
| No | 985 | 57.8 |  |  |
|  | ***Mean*** | **SD** | **Skewness** | **Kurtosis** |
| *Stressors scale – total score (0-100)* | 43.9 | 20.0 | 0.06 | -0.50 |
| Restrictions in daily life (0-100) | 63.7 | 23.5 | -0.55 | -0.29 |
| Under pressure/stressed (0-100) | 54.6 | 28.4 | -0.32 | -0.96 |
| Anxious/insecure (0-100) | 38.7 | 28.6 | 0.34 | -1.00 |
| Loneliness/social isolation (0-100) | 46.8 | 30.9 | 0.04 | -1.19 |
| Financial-economic burden (0-100) | 16.0 | 25.0 | 1.77 | 2.36 |
| *WHO-5 wellbeing (0-100)* | 45.0 | 19.7 | 0.16 | -0.60 |
| *BLMSS-12 Life satisfaction (0-100)* | 66.0 | 16.0 | -0.52 | 0.05 |
| *Gratitude/Awe-7 (0-100)* | 52.7 | 17.6 | 0.07 | 0.12 |
| *Perceived changes Questionnaire (0-100)* |  |  |  |  |
| Nature/silence/contemplation | 48.1 | 19.7 | -0.08 | -0.21 |
| Spirituality | 19.9 | 23.3 | 1.03 | 0.09 |
| Relationships | 59.3 | 20.5 | -0.32 | 0.10 |
| Reflection on life | 54.8 | 24.7 | -0.32 | -0.53 |
| Digital media usage | 54.5 | 24.3 | -0.15 | -0.59 |
| Restrictions | 58.9 | 22.3 | -0.34 | -0.50 |
| Memento mori | 45.2 | 22.2 | 0.21 | -0.68 |

Abbreviations: *BLMSS* Brief Multidimensional Life Satisfaction Scale, *SD* standard deviation, *WHO* World Health Organization

**Online Appendix 2: German Translation of the Death Reflection Scale**

Das Erleben der Corona-Pandemie kann durch die Konfrontation mit Krankheit und durch persönliche Betroffenheit einer schwer oder tödlich verlaufenden Infektionserkrankung mit dem Coronavirus oder den in den Medien berichteten Todesfällen unsere Einstellung zu unserer eigenen Sterblichkeit verändern.

Wir möchten Sie deshalb fragen, ob Sie seit Beginn der Corona-Pandemie anders über den Tod und das Sterben nachdenken.

Wie sehr stimmen Sie den folgenden Aussagen zu?

| Wenn ich über den Tod nachdenke, dann… | Ich stimme überhaupt nicht zu. - - - - - Ich stimme voll und ganz zu. |
| --- | --- |
| 1 … denke ich, dass ich mehr für die Welt tun sollte. |  |
| 2 … fühle ich einen starken Drang, anderen Menschen zu helfen. |  |
| 3 … möchte ich großzügiger oder selbstloser sein. |  |
| 4 … mache ich Pläne für mein Leben. |  |
| 5 … denke ich über die Dinge nach, die ich noch tun will. |  |
| 6 … bin ich motiviert, neue Dinge auszuprobieren. |  |
| 7 … kann ich kleine Probleme loslassen. |  |
| 8 … zerbreche ich mir wegen Kleinigkeiten nicht mehr den Kopf. |  |
| 9 … fühle ich mich weniger gestresst aufgrund der Dinge, die mir etwas ausmachen. |  |
| 10 … denke ich darüber nach, was ich der Welt hinterlasse. |  |
| 11 … denke ich darüber nach, was andere Menschen wohl von mir nach meinem Tod denken. |  |
| 12 … denke ich darüber nach, wie ich wohl anderen Menschen in Erinnerung bleibe. |  |
| 13 … möchte ich mehr Zeit mit den Menschen verbringen, die wichtig für mich sind. |  |
| 14 … möchte ich vermehr Menschen, die mir wichtig sind, mitteilen, wie viel sie mir bedeuten. |  |
| 15 … möchte ich mehr Zeit mit meiner Familie verbringen. |  |

**Online Appendix 3: Subscale distributions of the Death Reflection Scale among age and occupational groups**

**Online Figure 1** Subscale distributions of the Death Reflection Scale among age groups

| 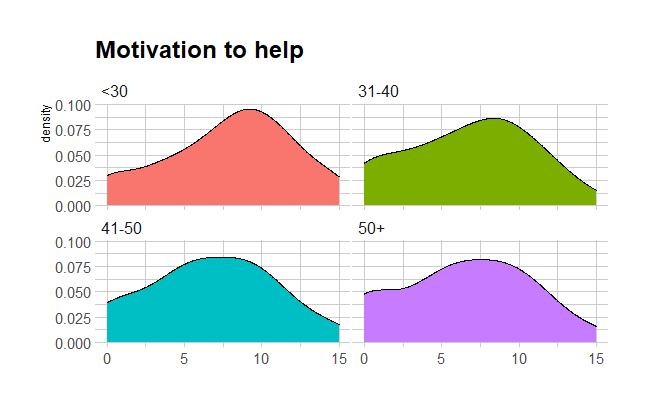 | 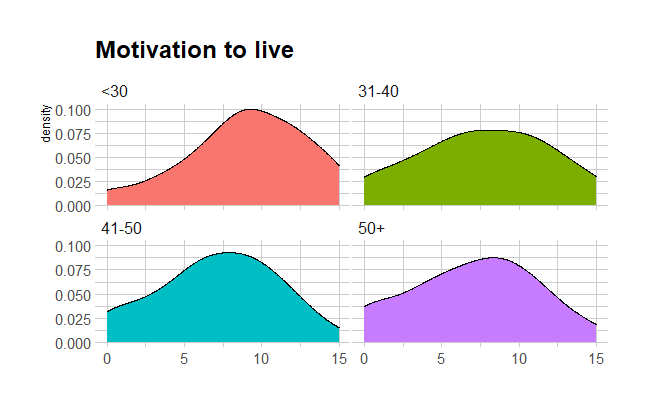 |
| --- | --- |
| 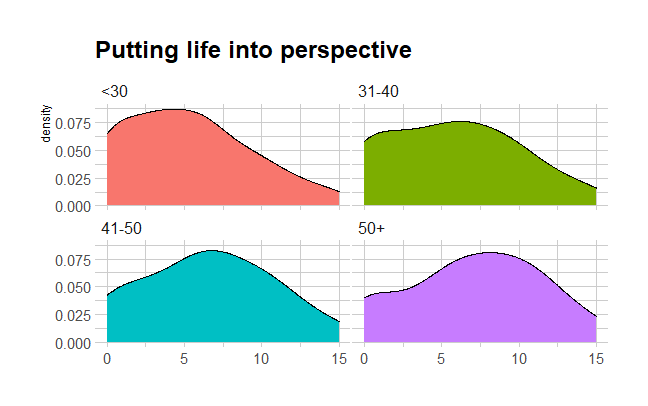 | 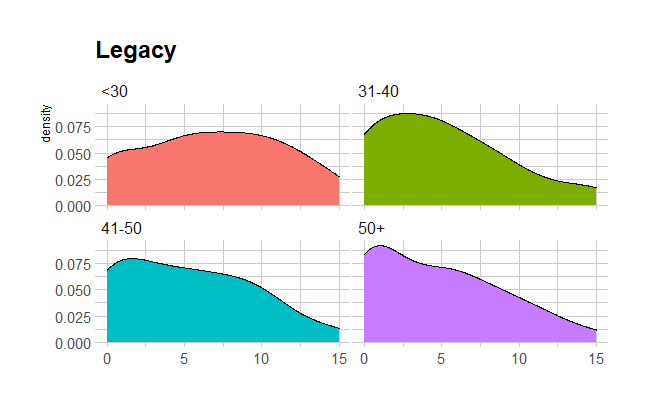 |
| 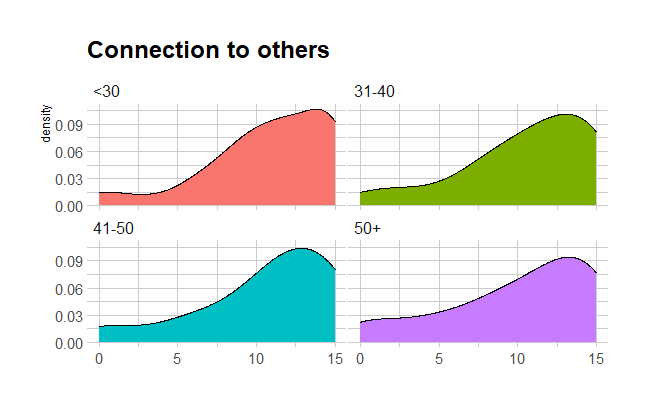 |  |

**Online Figure 2** Subscale distributions of the Death Reflection Scale among occupational groups


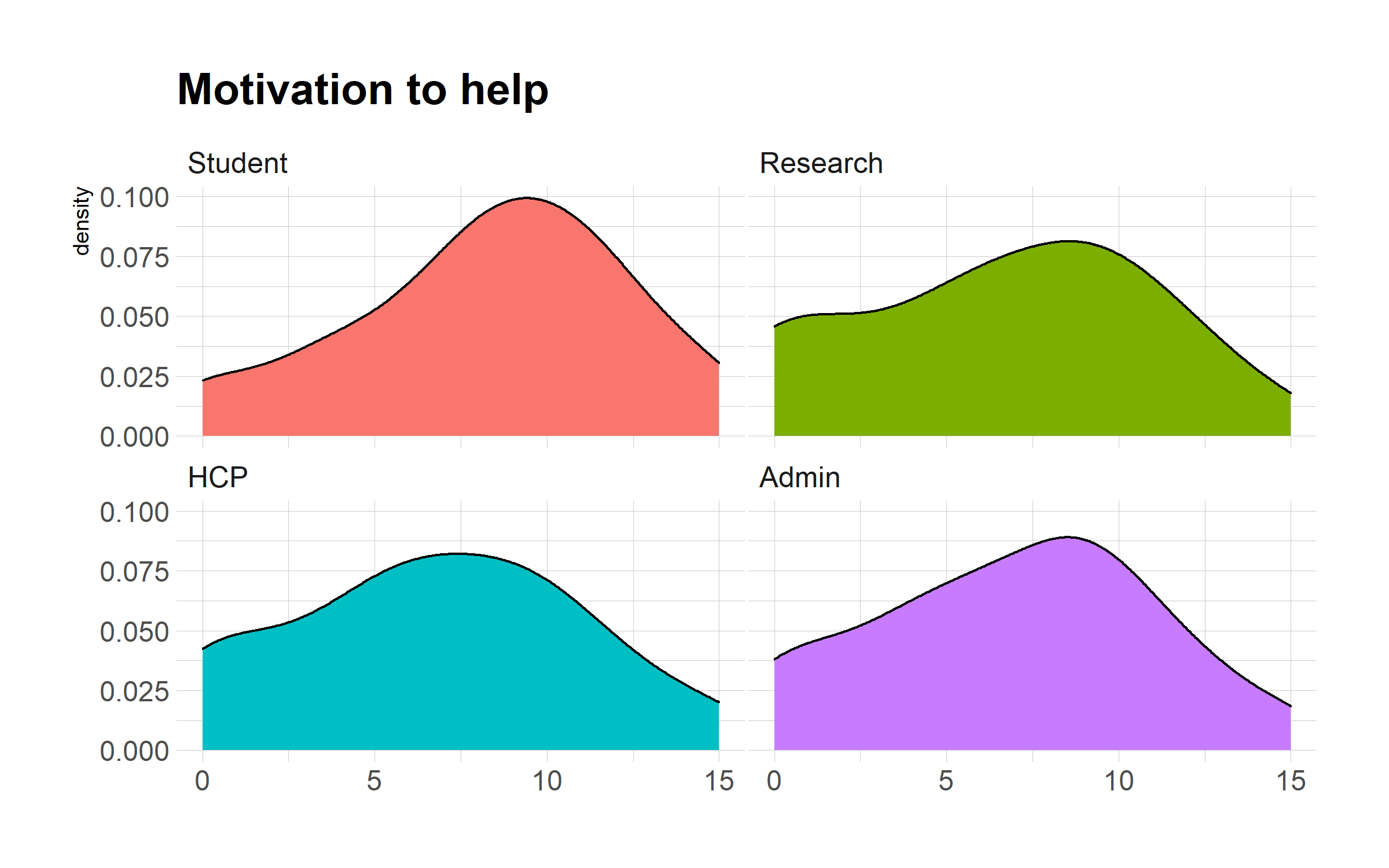

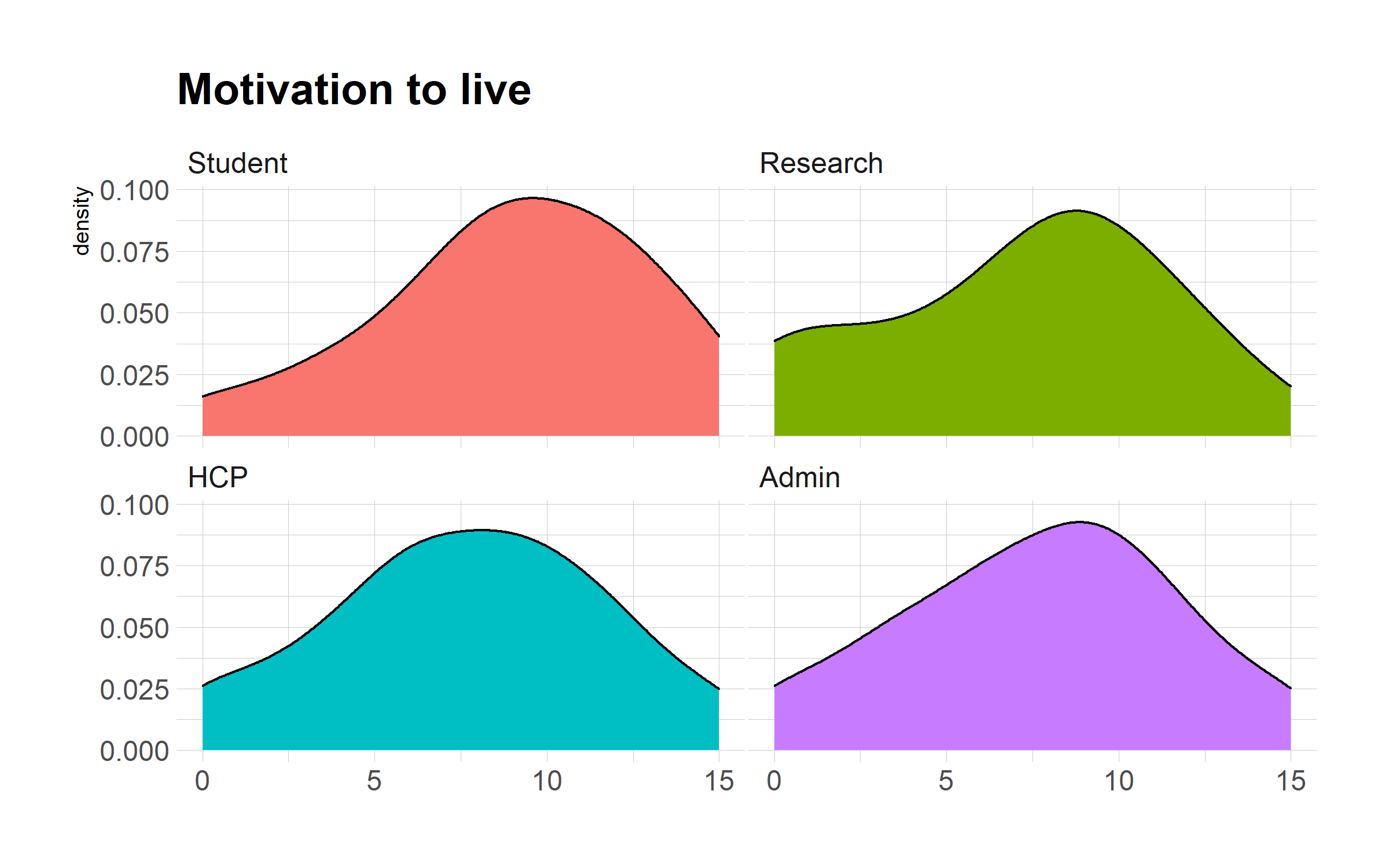

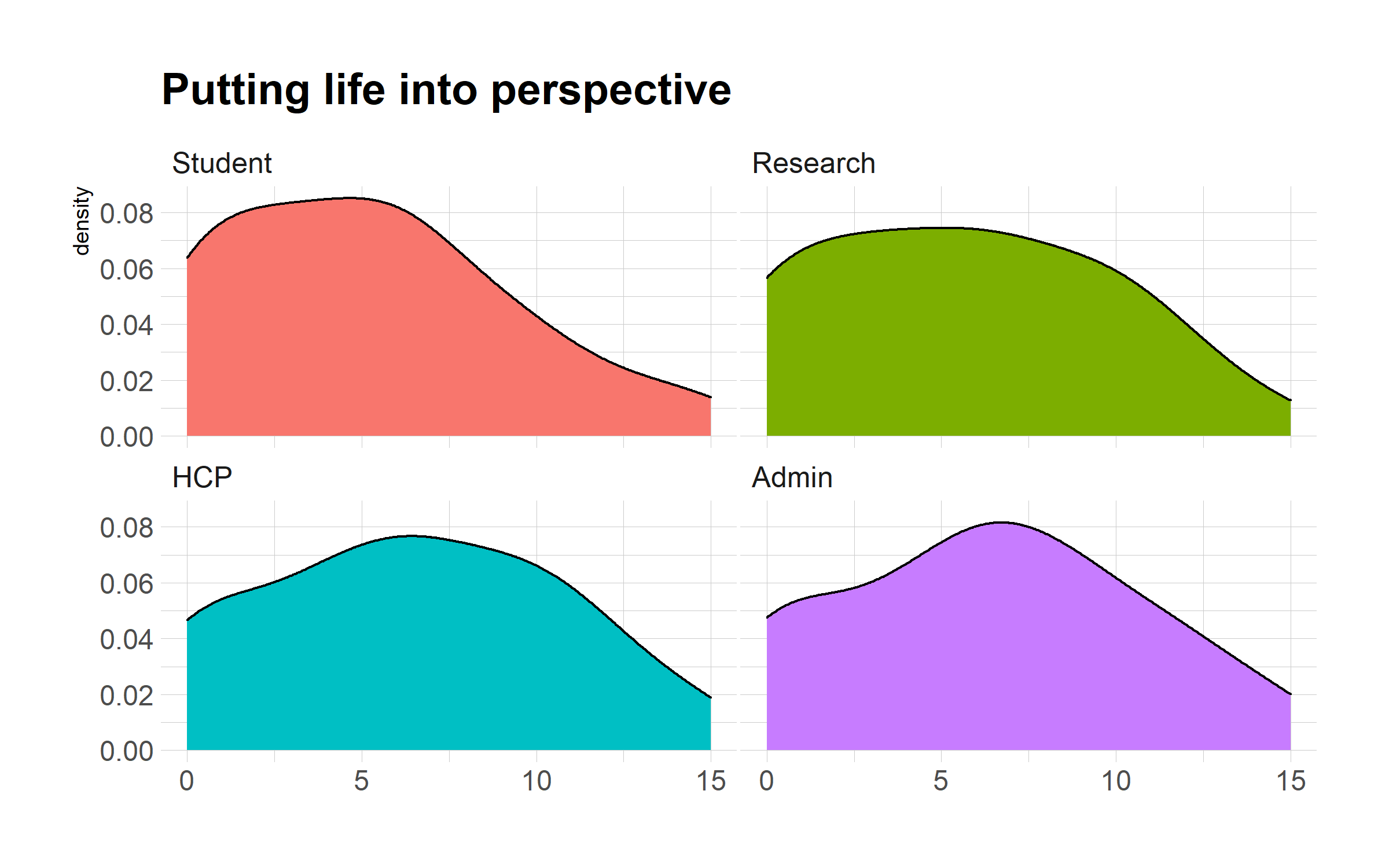

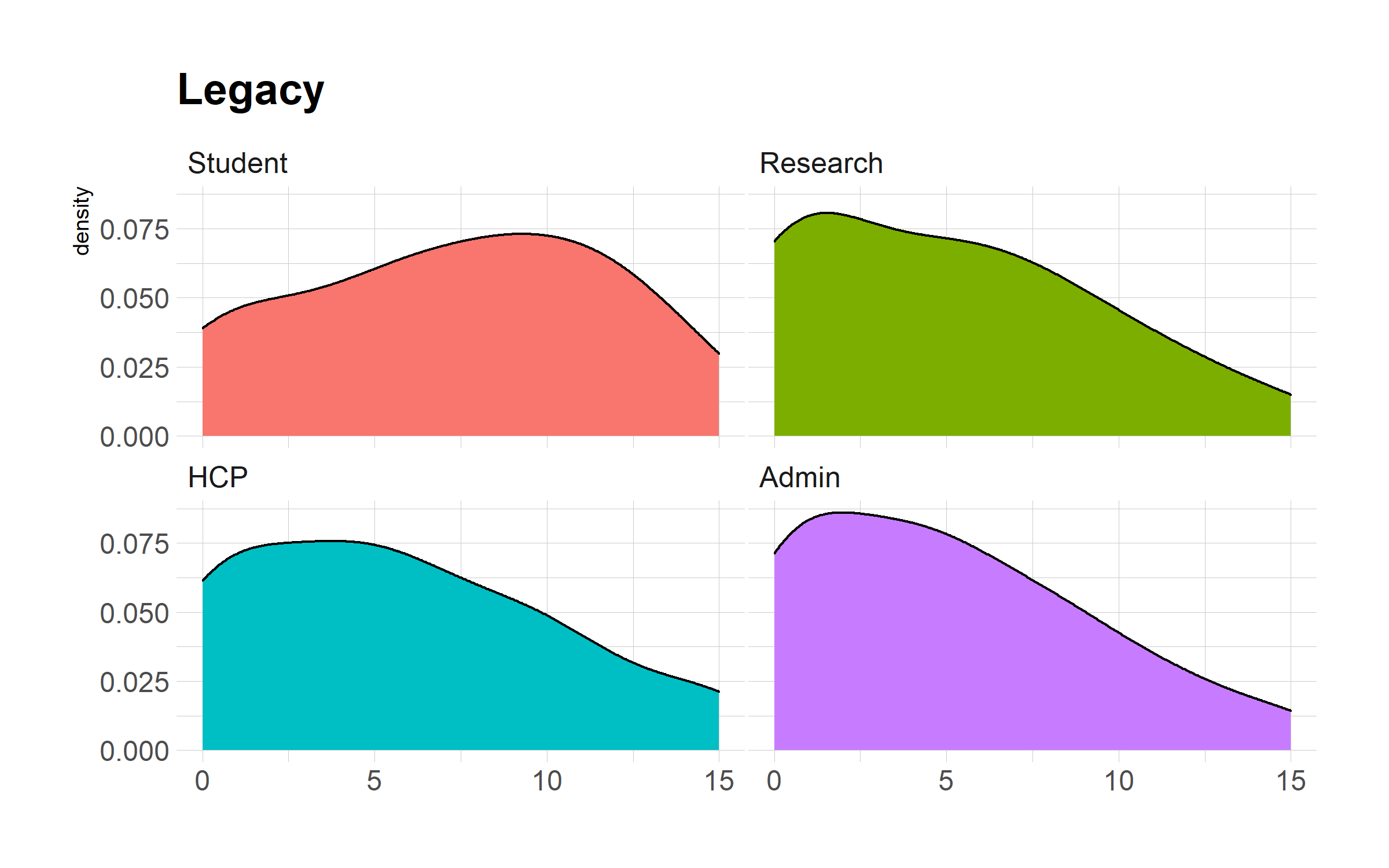

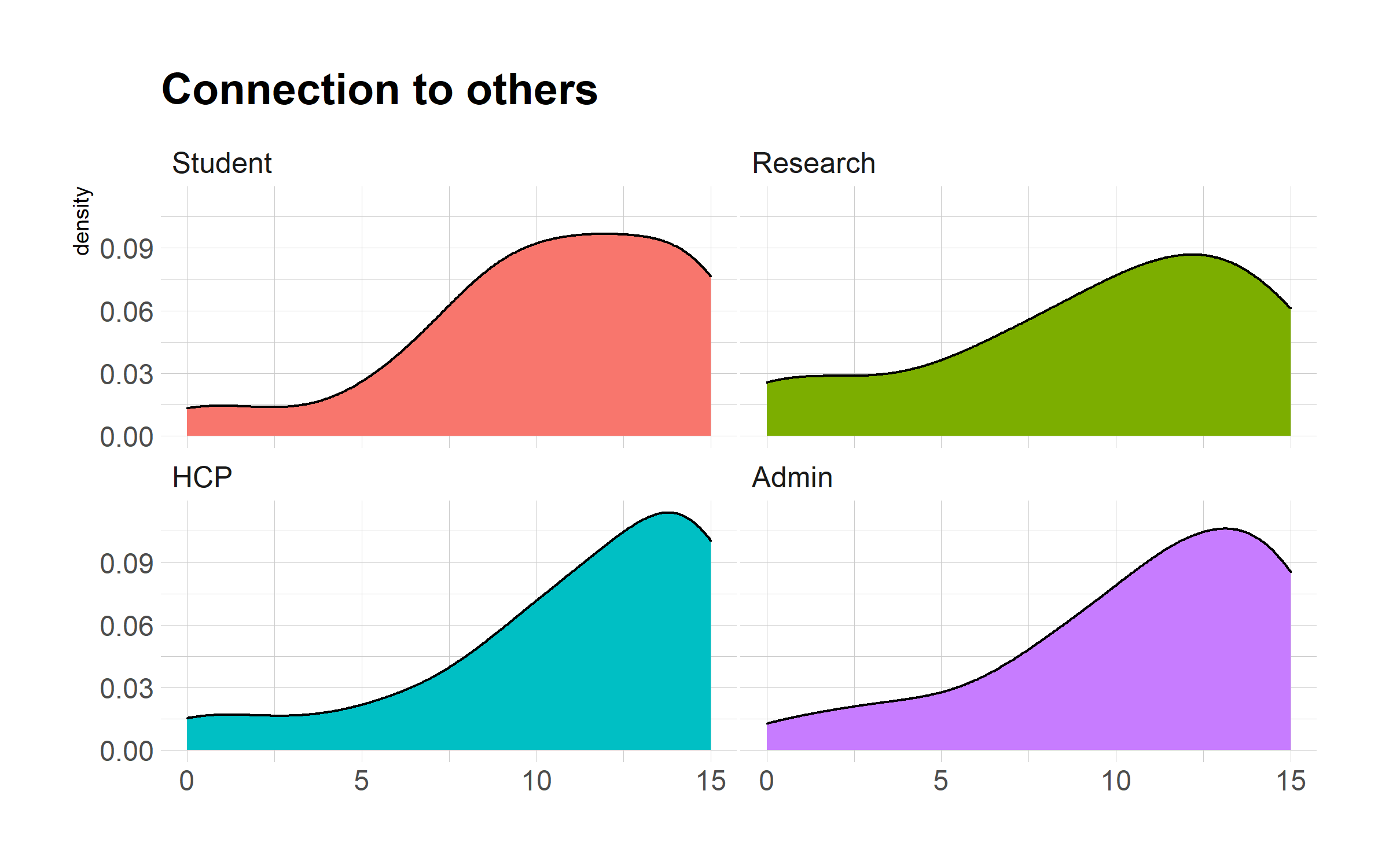


Abbreviations: HCP – health care professional, Admin – administration/library/IT services

**Online Appendix 4: Scale/Subscale Means and Standard deviations (SDs) by Age group**

|  | **Age group** |  |  |  |  |  |  |
| --- | --- | --- | --- | --- | --- | --- | --- |
|  | **<30 years** | **31-40 years** | **41-50 years** | **51-60 years** | **>60 years** | **Total sample** |  |
|  | ***M (SD)*** | ***M (SD)*** | ***M (SD)*** | ***M (SD)*** | ***M (SD)*** | ***M (SD)*** | ***p*** |
| *N* | 631 | 346 | 282 | 342 | 102 | 1,703 |  |
| Sex |  |  |  |  |  |  |  |
| Men | 171 (24.6) | 124 (31.9) | 76 (24.3) | 80 (21.4) | 35 (32.3) | 697 (36.9) | .276 |
| Women | 523 (75.1) | 263 (67.7) | 236 (75.7) | 294 (78.5) | 73 (67.7) | 389 (20.6) |  |
| Death Reflection Scale |  |  |  |  |  |  |  |
| Motivation to help | 7.92 (4.17) | 6.79 (4.05) | 6.59 (4.04) | 6.50 (4.19) | 6.14 (4.08) | 7.05 (4.17) | .000 |
| Motivation to live | 8.94 (3.93) | 7.73 (4.22) | 6.95 (3.82) | 6.99 (4.07) | 6.79 (4.41) | 7.82 (4.12) | .000 |
| Putting life into perspective | 5.32 (4.07) | 5.80 (4.22) | 6.69 (4.21) | 7.13 (4.26) | 7.32 (4.43) | 6.15 (4.25) | .000 |
| Legacy | 6.89 (4.59) | 5.04 (4.18) | 5.22 (4.27) | 4.68 (4.32) | 5.06 (4.04) | 5.65 (4.45) | .000 |
| Connection to others | 10.70 (4.04) | 10.42 (4.14) | 10.25 (4.39) | 9.87 (4.60) | 10.16 (5.03) | 10.37 (4.30) | .496 |
| Life Satisfaction | 65.50 (15.84) | 64.68 (15.96) | 66.23 (16.12) | 67.94 (15.91) | 64.40 (17.23) | 65.88 (16.00) | .115 |
| WHO-5 well-being | 43.53 (18.05) | 44.76 (19.10) | 44.73 (20.13) | 46.73 (21.30) | 49.30 (20.75) | 44.98 (19.51) | .326 |
| Gratitude/Awe-7 | 51.21 (16.99) | 48.38 (16.79) | 52.82 (17.84) | 57.87 (18.12) | 56.09 (18.39) | 52.53 (17.67) | .000 |
| Stressors | 49.18 (19.16) | 44.02 (19.14) | 40.86 (19.53) | 39.02 (19.23) | 36.86 (21.27) | 43.86 (19.81) | .000 |
| Relationships | 59.24 (20.43) | 56..54 (20.23) | 59.23 (20.17) | 60.71 (20.74) | 55.73 (20.86) | 58.79 (20.44) | .407 |
| Reflection on life | 58.96 (23.75) | 50.07 (24.74) | 51.38 (24.11) | 54.32 (25.59) | 58.44 (24.05) | 54.87 (24.64) | .000 |
| Restrictions in life | 64.18 (20.48) | 58.26 (22.45) | 55.40 (22.88) | 55.20 (23.43) | 53.05 (26.96) | 58.96 (22.51) | .000 |
| Memento mori module | 43.11 (21.52) | 39.97 (22.10) | 45.42 (22.69) | 50.26 (22.61) | 55.44 (21.11) | 45.10 (22.25) | .000 |

Abbreviations: *WHO* World Health Organization

**Online Appendix 5: Scale/Subscale Means and Standard deviations (SDs) by Occupational group**

|  | **Occupational group** |  |  |  |  |  |
| --- | --- | --- | --- | --- | --- | --- |
|  | **Student** | **Researcher** | **HCP** | **Administration** | **Total sample** |  |
|  | ***M (SD)*** | ***M (SD)*** | ***M (SD)*** | ***M (SD)*** | ***M (SD)*** | ***p*** |
| *N* | 372 | 287 | 407 | 426 | 1,492 |  |
| Sex (*n*, %) |  |  |  |  |  |  |
| Men | 117 (31.5) | 109 (37.9) | 76 (18.7) | 97 (22.8) | 399 (26.7) | .000 |
| Women | 255 (68.5) | 178 (62.1) | 331 (81.3) | 329 (77.2) | 1093 (73.3) |  |
| Death Reflection Scale |  |  |  |  |  |  |
| Motivation to help | 8.47 (3.97) | 6.52 (4.25) | 6.74 (4.21) | 7.02 (4.03) | 7.19 (4.17) | .000 |
| Motivation to live | 9.01 (3.85) | 7.10 (4.20) | 7.58 (3.96) | 7.65 (3.97) | 7.90 (4.01) | .000 |
| Putting life into perspective | 5.29 (4.06) | 5.76 (4.22) | 6.50 (4.25) | 6.49 (4.30) | 6.03 (4.22) | .000 |
| Legacy | 7.48 (4.49) | 5.09 (4.34) | 5.74 (4.53) | 5.11 (4.23) | 5.87 (4.47) | .000 |
| Connection to others | 10.56 (3.93) | 9.26 (4.66) | 10.85 (4.30) | 10.60 (4.08) | 10.45 (4.20) | .000 |
| Life Satisfaction | 63.85 (15.94) | 67.12 (16.07) | 66.15 (15.22) | 65.69 (15.88) | 65.99 (15.82) | .029 |
| WHO-5 well-being | 43.66 (18.10) | 47.18 (18.65) | 45.97 (19.56) | 43.51 (20.60) | 45.05 (19.57) | .000 |
| Gratitude/Awe-7 | 53.37 (17.32) | 49.09 (17.49) | 52.79 (18.09) | 53.70 (18.11) | 52.69 (17.69) | .051 |
| Stressors | 52.62 (19.33) | 40.08 (18.33) | 41.69 (19.81) | 43.58 (19.29) | 44.39 (19.98) | .000 |
| Relationships | 60.36 (20.04) | 54.13 (21.37) | 59.48 (19.58) | 59.10 (20.20) | 59.01 (20.55) | .006 |
| Reflection on life | 61.33 (24.07) | 48.69 (24.65) | 54.21 (24.55) | 55.23 (25.54) | 54.92 (24.90) | .000 |
| Restrictions in life | 66.48 (20.97) | 53.93 (22.58) | 57.86 (21.41) | 59.25 (22.20) | 59.19 (22.13) | .000 |
| Memento mori module | 44.57 (22.68) | 40.09 (20.96) | 45.60 (22.31) | 48.49 (22.61) | 44.97 (22.28) | .000 |

Abbreviations: *HCP* healthcare professional, *M* mean, *N* sample size, *SD* standard deviation, *WHO* World Health Organization

**Online Appendix 6: Factor loading diagrams of four different baseline measurement models for the Death Reflection Scale**

**a) Unidimensional model**

|  | *X²*(*df*) | RMSEA | [90% CI] | CFI | SRMR | AIC | BIC |
| --- | --- | --- | --- | --- | --- | --- | --- |
| Unidimensional model | 4242 (90)* | 0.165 | 0.160, 0.169 | 0.407 | 0.147 | 88682 | 88845 |

*X² test statistically significant


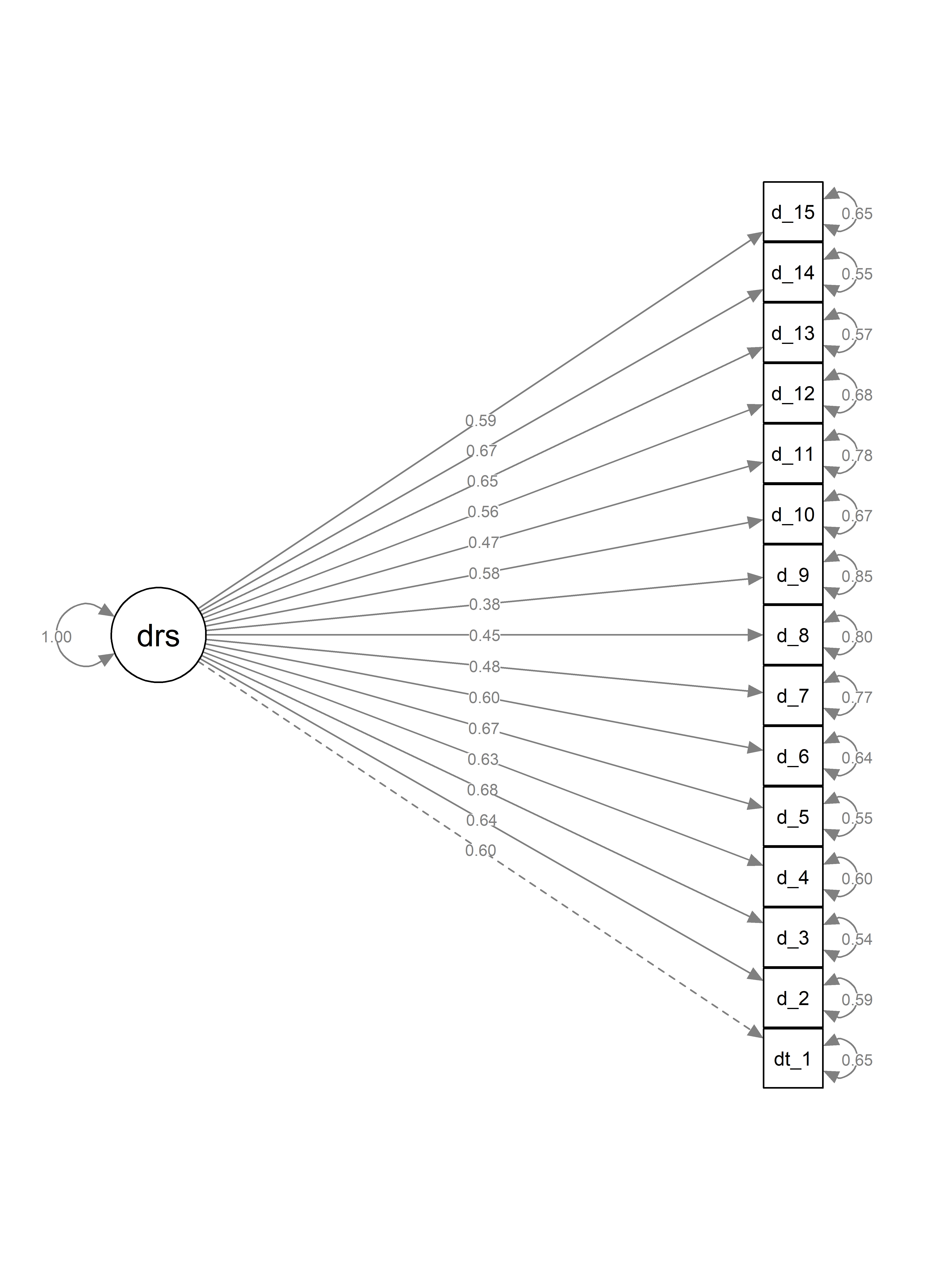


Abbreviations: DRS: Death reflection scale, d_1: I feel like I should do more for the world; d_2: I feel a strong urge to help other people; d_3: I want to be a more generous person, d_4: I make plans for my life; d_5: I reflect on the things I still want to do, d_6: I am motivated to try new things; d_7: I can let go of the little problems; d_8: I am able to stop sweating the small stuff; d_9: I am less stressed about the things that are bothering me; d_10: I think about what legacy I will have left behind; d_11: I reflect on whether people will think of me after death; d_12: I reflect on how I will be remembered, d_13: I want to spend more time with the people I care about; d_14: I want to tell the people I care about how I feel about them; d_15: I want to spend more time with my family.

**b) 5-factor model as proposed in Yuan et al. (2019)**

|  | *X²*(*df*) | RMSEA | [90% CI] | CFI | SRMR | AIC | BIC |
| --- | --- | --- | --- | --- | --- | --- | --- |
| 5-factor model | 598.2 (80)* | 0.062 | 0.057, 0.066 | 0.926 | 0.039 | 80927 | 81144 |

*X² test statistically significant


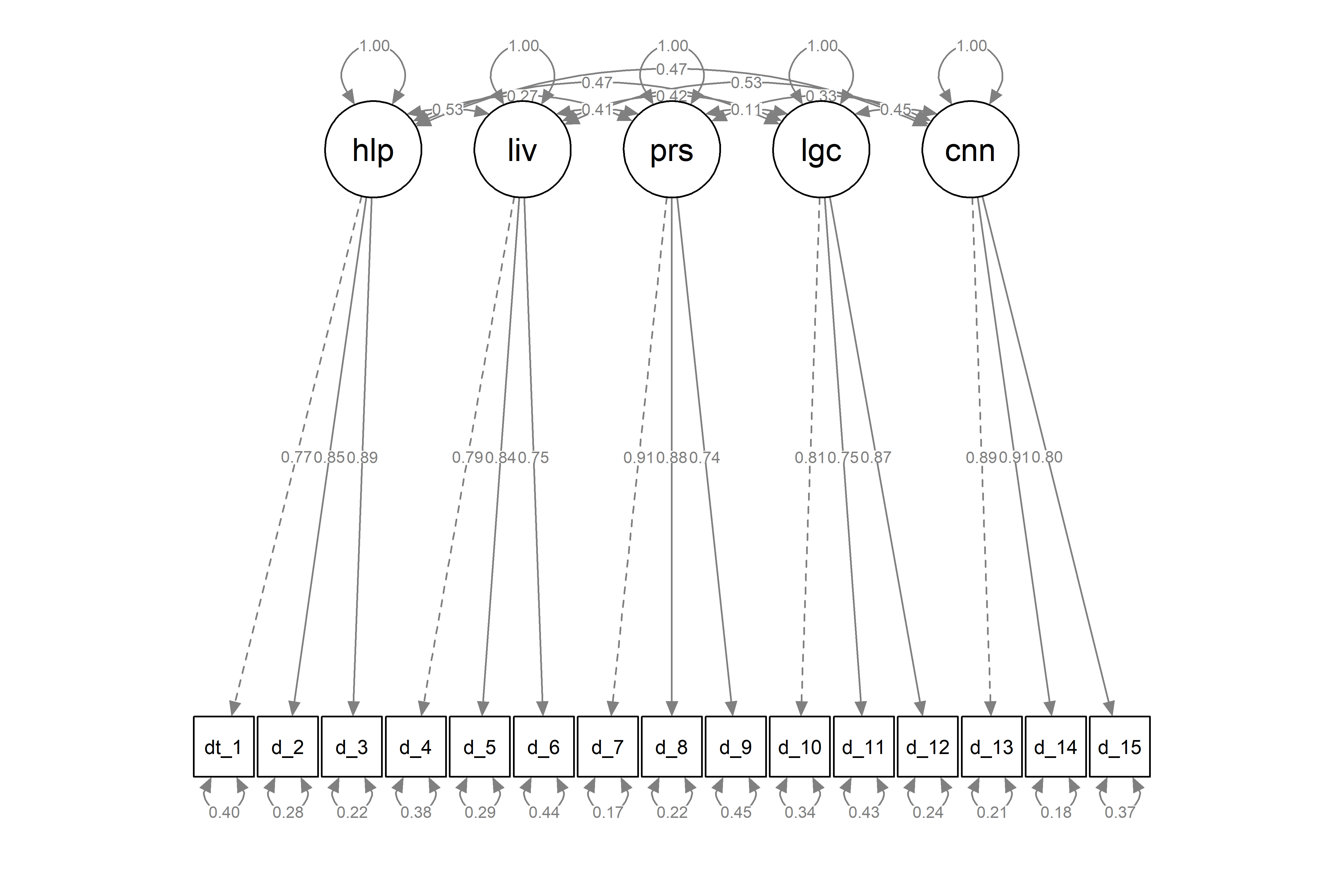


Abbreviations: DRS: Death reflection scale, d_1: I feel like I should do more for the world; d_2: I feel a strong urge to help other people; d_3: I want to be a more generous person, d_4: I make plans for my life; d_5: I reflect on the things I still want to do, d_6: I am motivated to try new things; d_7: I can let go of the little problems; d_8: I am able to stop sweating the small stuff; d_9: I am less stressed about the things that are bothering me; d_10: I think about what legacy I will have left behind; d_11: I reflect on whether people will think of me after death; d_12: I reflect on how I will be remembered, d_13: I want to spend more time with the people I care about; d_14: I want to tell the people I care about how I feel about them; d_15: I want to spend more time with my family; hlp: Factor 1 - Motivation to help, liv: Factor 2 – Motivation to live, prs: Factor 3 – Putting life into perspective, lgc: Factor 4 – Legacy, cnn: Factor 5 – Connection to others.

**c) Second-order model of the DRS**

|  | *X²*(*df*) | RMSEA | [90% CI] | CFI | SRMR | AIC | BIC |
| --- | --- | --- | --- | --- | --- | --- | --- |
| 5-factor model | 618.4 (85)* | 0.061 | 0.056, 0.065 | 0.924 | 0.051 | 80990 | 81181 |

*X² test statistically significant


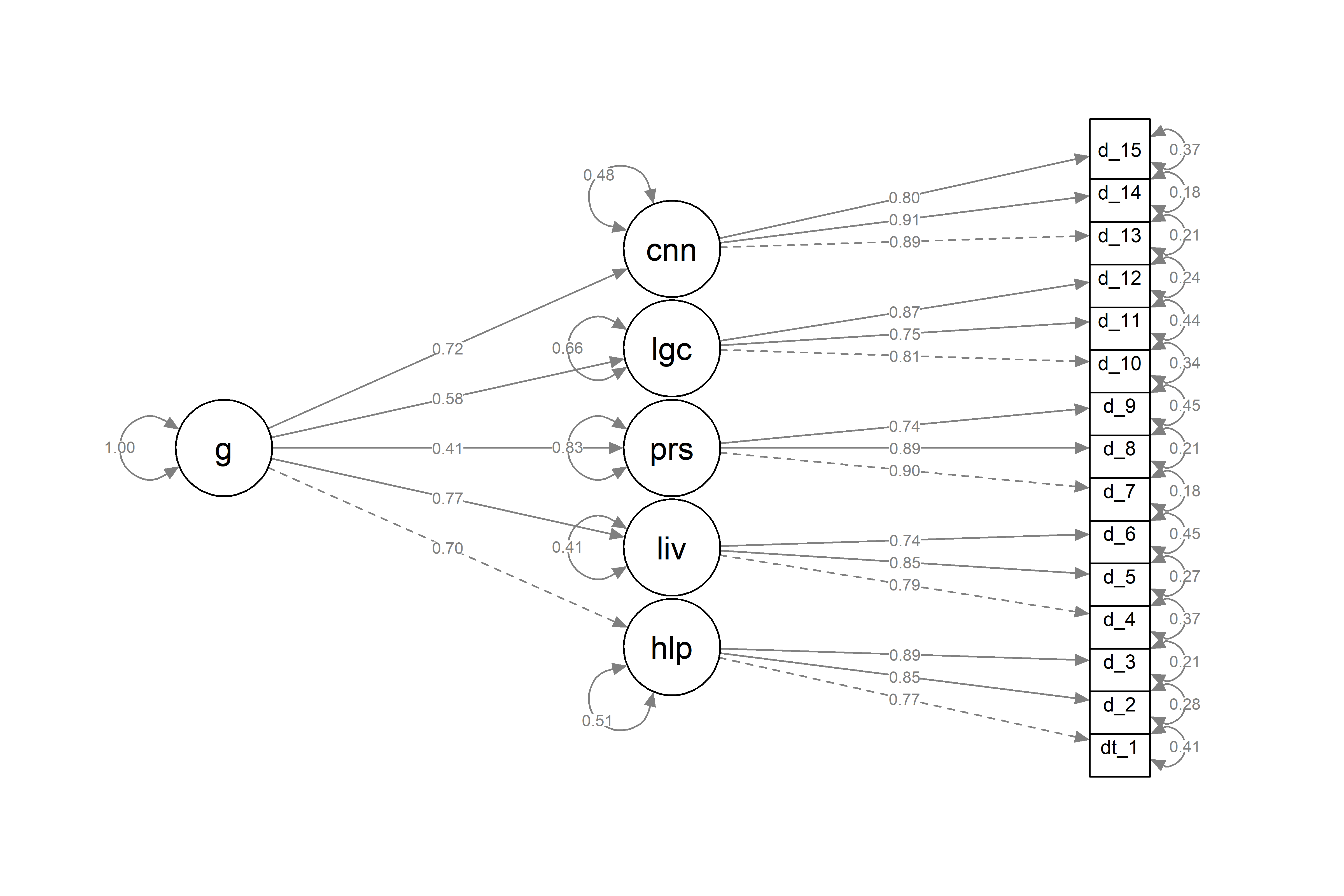


Abbreviations: DRS: Death reflection scale, d_1: I feel like I should do more for the world; d_2: I feel a strong urge to help other people; d_3: I want to be a more generous person, d_4: I make plans for my life; d_5: I reflect on the things I still want to do, d_6: I am motivated to try new things; d_7: I can let go of the little problems; d_8: I am able to stop sweating the small stuff; d_9: I am less stressed about the things that are bothering me; d_10: I think about what legacy I will have left behind; d_11: I reflect on whether people will think of me after death; d_12: I reflect on how I will be remembered, d_13: I want to spend more time with the people I care about; d_14: I want to tell the people I care about how I feel about them; d_15: I want to spend more time with my family; hlp: Factor 1 - Motivation to help, liv: Factor 2 – Motivation to live, prs: Factor 3 – Putting life into perspective, lgc: Factor 4 – Legacy, cnn: Factor 5 – Connection to others, g: general factor.

**d) Hierarchical (bifactor) model of the DRS**

|  | *X²*(*df*) | RMSEA | [90% CI] | CFI | SRMR | AIC | BIC |
| --- | --- | --- | --- | --- | --- | --- | --- |
| Higher-order model  (Bifactor model) | 463.4 (75)* | 0.055 | 0.050, 0.060 | 0.945 | 0.045 | 80814 | 81058 |

*X² test statistically significant


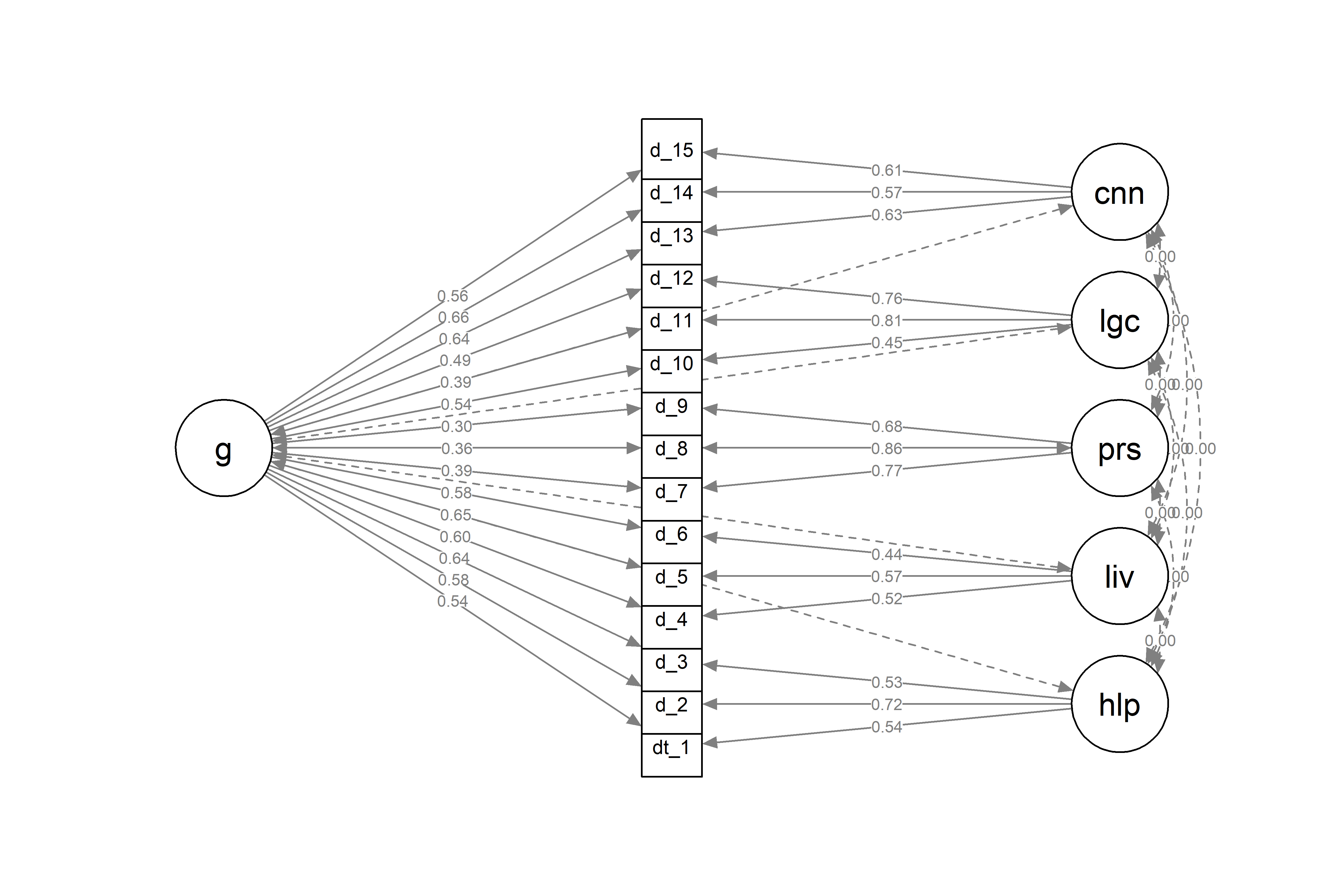


Abbreviations: DRS: Death reflection scale, d_1: I feel like I should do more for the world; d_2: I feel a strong urge to help other people; d_3: I want to be a more generous person, d_4: I make plans for my life; d_5: I reflect on the things I still want to do, d_6: I am motivated to try new things; d_7: I can let go of the little problems; d_8: I am able to stop sweating the small stuff; d_9: I am less stressed about the things that are bothering me; d_10: I think about what legacy I will have left behind; d_11: I reflect on whether people will think of me after death; d_12: I reflect on how I will be remembered, d_13: I want to spend more time with the people I care about; d_14: I want to tell the people I care about how I feel about them; d_15: I want to spend more time with my family; hlp: Factor 1 - Motivation to help, liv: Factor 2 – Motivation to live, prs: Factor 3 – Putting life into perspective, lgc: Factor 4 – Legacy, cnn: Factor 5 – Connection to others, g: general factor.
